# Druggable genome in attention deficit/hyperactivity disorder and its co-morbid conditions. New avenues for treatment

**DOI:** 10.1038/s41380-019-0540-z

**Published:** 2019-10-18

**Authors:** Tor-Arne Hegvik, Kai Waløen, Sunil K. Pandey, Stephen V. Faraone, Jan Haavik, Tetyana Zayats

**Affiliations:** 1 K.G. Jebsen Centre for Research on Neuropsychiatric Disorders, Department of Biomedicine, University of Bergen, Bergen, Norway; 2 Departments of Psychiatry and of Neuroscience and Physiology, SUNY Upstate Medical University, Syracuse, NY, USA; 3 Division of Psychiatry, Haukeland University Hospital, Bergen, Norway; 4 Stanley Center for Psychiatric Research, Broad Institute of MIT and Harvard, Cambridge, MA, USA; 5 Analytic and Translational Genetics Unit, Department of Medicine, Massachusetts General Hospital, Boston, MA, USA

## Abstract

Attention-Deficit/Hyperactivity Disorder (ADHD) is a common neurodevelopmental disorder with only symptomatic care available. Genome-wide association (GWA) studies can provide a starting point in the search for novel drug targets and possibilities of drug repurposing. Here, we explored the druggable genome in ADHD by utilising GWA studies on ADHD and its co-morbid conditions. First, we explored whether the genes targeted by current ADHD drugs show association with the disorder and/or its co-morbidities. Second, we aimed to identify genes and pathways involved in the biological processes underlying ADHD that can be targeted by pharmacological agents. These ADHD-associated druggable genes and pathways were also examined in co-morbidities of ADHD, as commonalities in their aetiology and management may lead to novel pharmacological insights. Strikingly, none of the genes encoding targets of first-line pharmacotherapeutics for ADHD were significantly associated with the disorder, suggesting that FDA-approved ADHD drugs may act through different mechanisms than those underlying ADHD. In the examined druggable genome, three loci on chromosomes 1, 4 and 12 revealed significant association with ADHD and contained nine druggable genes, five of which encode established drug targets for malignancies, autoimmune and neurodevelopmental disorders. To conclude, we present a framework to assess the druggable genome in a disorder, exemplified by ADHD. We highlight signal transduction and cell adhesion as potential novel avenues for ADHD treatment. Our findings add to knowledge on known ADHD drugs and present the exploration of druggable genome associated with ADHD, which may offer interventions at the aetiological level of the disorder.

## Introduction

Attention deficit/hyperactivity disorder (ADHD) is a common and highly heritable childhood-onset neurodevelopmental disorder that often persists into adulthood [1, 2]. The prevalence of the disorder in children is 6.5%, while in adults the estimates vary between 2.5 and 3.4% [3]. ADHD patients are at high risk of experiencing difficulties in their education and social integration [4], elevated rates of incarceration, unemployment and accidental deaths, all resulting in high societal and economic burden [1, 5–7]. To date, no treatments cure ADHD, although available therapies offer symptomatic relief.

Current management of ADHD is based on either non-pharmacologic or pharmacologic treatments as well as the combination of the two. The non-pharmacologic treatments usually involve psychological and/or behavioural therapies, while the pharmacologic interventions include stimulant and/or non-stimulant drugs [1, 8]. For ADHD treatment, the U.S. Food and Drug Administration (FDA) has approved the stimulants methylphenidate (MPH) and amphetamine (AMP) along with three non-stimulants: atomoxetine, clonidine and guanfacine [9]. In many guidelines, MPH and AMP are the first-line agents for ADHD pharmacotherapy, exerting their primary effect by increasing dopamine and norepinephrine activity [10, 11]. Atomoxetine is a selective norepinephrine re-uptake inhibitor, while both clonidine and guanfacine are alpha-2 adrenoreceptor agonists [11]. Although all the pharmaceuticals used to manage ADHD are believed to act on biological pathways underlying the disorder, their complete mechanisms of action remain unknown, as are the causal biological mechanisms of ADHD.

An important feature of ADHD relevant to the search of new medications is the existence of co-morbid conditions [12, 13]. As it has been postulated that biological processes underlying ADHD may also be involved in the development of its co-morbidities [14], it is prudent to examine the relationship between ADHD-associated druggable loci and those conditions. The exploration of associations between a gene encoding or being the target of a drug and a number of phenotypes has been proposed to aid pharmacotherapeutics by capturing a broader spectrum of relevant biological information and offering alternatives to existing pharmaceuticals to treat a disorder (drug repurposing) [15].

Randomised controlled studies have shown that pharmacotherapy reduces the ADHD symptom burden [16] and observational studies have reported that it improves important life outcomes, such as academic performance [17], social functioning [18, 19] and the rate of motor vehicle accidents [20]. Nonetheless, the current pharmacological treatment of ADHD is not curative and, although many patients improve markedly, optimal outcomes are difficult to achieve, especially with regards to signs of executive dysfunction and emotional dysregulation [21, 22]. There are also lingering concerns about long-term effects of stimulants on growth and weight [23–25]. Thus, there is a need for more efficient and safe pharmacological agents to treat and, eventually, cure ADHD.

Improvements in the pharmacotherapeutic options for ADHD may require a fuller understanding of its underlying biological processes [26]. As knowledge on the genetics of common disorders evolves, novel strategies for the development of new and improved pharmacotherapeutics are emerging. For complex disorders, such as ADHD, genomewide association (GWA) studies can uncover genes and pathways involved in the disease aetiology, yielding innovative avenues for future drug development and repurposing [27, 28].

In this study, we explored the druggable genome in ADHD by utilising the summary statistics from GWA studies on ADHD and its major co-morbid conditions. We aimed to (1) explore whether the genes targeted by current FDA-approved ADHD drugs show association with the disorder and/or its co-morbidities, (2) identify genes and pathways involved in the biological processes underlying ADHD, its co-morbidities and quality of life phenotypes that can be targeted by pharmacological compounds and (3) examine the identified druggable genes and pathways as potential options for novel drug development and repurposing.

## Materials and methods

**Fig. 1:**
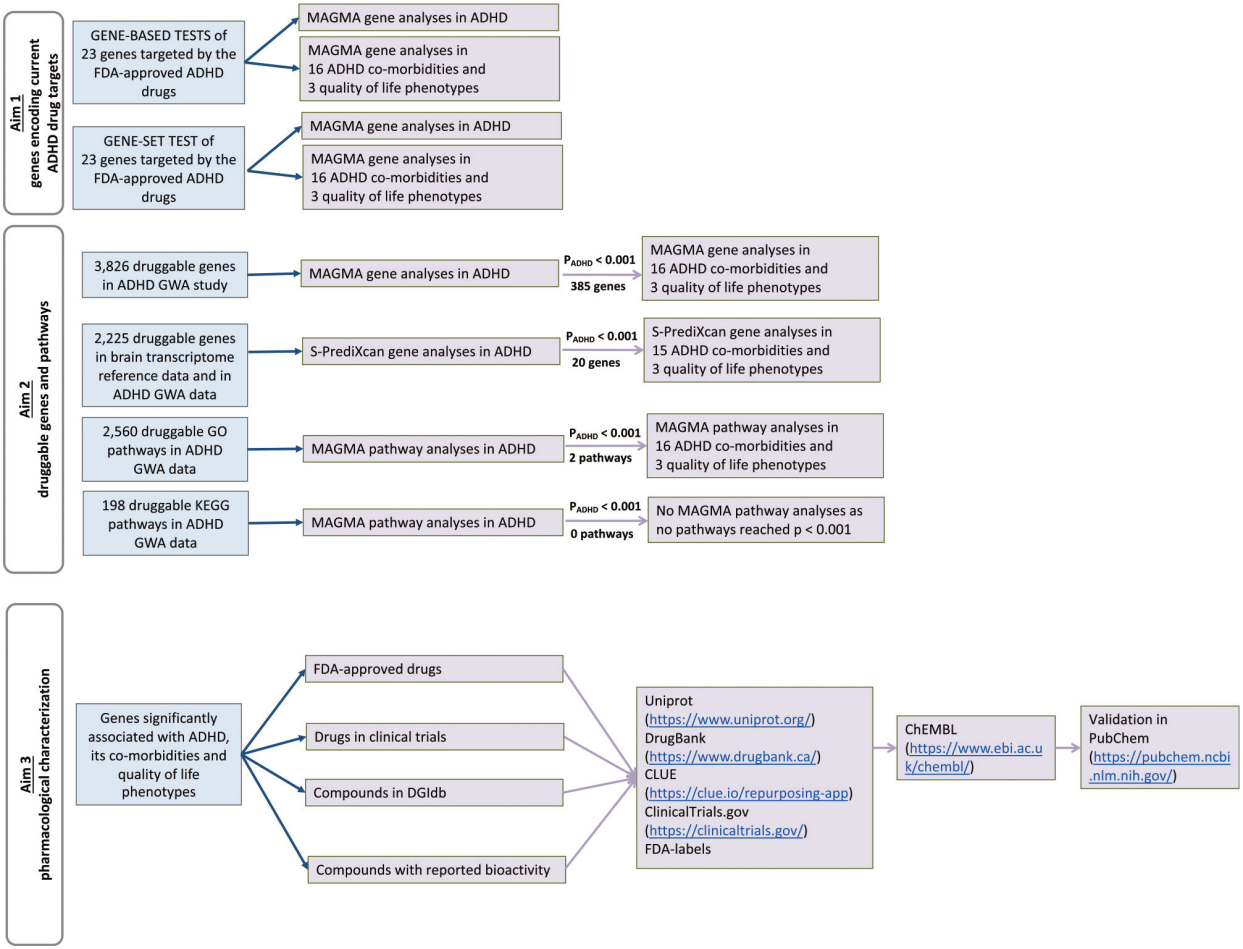
Study design flowchart. ADHD; attention deficit/hyperactivity disorder, BMI; body mass index, CHD; coronary heart disease, T2DM; type 2 diabetes mellitus, SWB; subjective well-being

Figure 1 shows a flowchart summarising the steps of our study.

### Definition of the druggable genome

The druggable genome was defined as described in Finan et al. [29] as a selection of genes that are potential targets for pharmacological intervention.

The identification of these druggable genes was based on the protein targets of known and experimental drugs, sequence similarities to those targets (potential druggability), drug–gene interactions, biotherapeutics and a number of databases documenting pharmacological molecules and their therapeutic targets.

### Definition of ADHD co-morbidities and quality of life phenotypes

For the purpose of this study, we focused only on common conditions with well-documented evidence for association with ADHD based on large-scale genetic [30] and epidemiological [31] studies, together with a systematic literature review [32] complemented by a PubMed search using the following criteria “((ADHD co-morbidity) AND English [Language]) AND (“2015” [Date-Publication]: “3000” [Date-Publication])”.

Additional criteria were the availability of large (≥20,000 individuals) GWA studies and their summary statistics. Thus, where such data were not available, we used proxy phenotypes (e.g. instead of insomnia disorder, we examined insomnia symptoms [33]).

We characterised the co-morbidities into the three main groups: (1) cardiometabolic, (2) immune-inflammatory-autoimmune (referred to as immune) and (3) neuropsychiatric.

In addition to well-defined clinical diagnoses, ADHD has been reported to be associated with reduced quality of life, reduced educational attainment and sleep disturbances [1]. Therefore, we also examined the druggable genome overlapping between ADHD and educational attainment, sleep duration and subjective well-being as proxies for quality of life and functional outcomes associated with ADHD.

### Genetic data

We relied on summary statistics derived from large-scale GWA studies. Where possible, we restricted our analyses to individuals of European descent only, meta-analysed sample size equal to or larger than 70% of the total sample, variants with minor allele frequency above or equal to 1% and of good imputation quality (INFO ≥ 0.8). For ADHD, summary statistics were acquired from the large-scale meta-analysis of 19,099 cases and 34,194 controls [30]. For the co-morbidities, we curated data from openly available resources or through correspondence with the authors of the GWA studies of interest (Table 1).

### Statistical analyses

Statistical analyses were divided into two main steps to address our first two aims: (1) examination of genes targeted by current FDA-approved ADHD drugs and (2) examination of the genes within the druggable genome and their pathways defined as known biological pathways containing at least one gene from the druggable genome. In step 1, we examined all genes in all curated GWA data. In step 2, we first examined ADHD and only genes and pathways that revealed suggestive association with it (*p* ≤ 0.001) were further analysed in the GWA data of its co-morbidities and quality of life phenotypes. We applied Bonferroni correction to account for multiple testing.

#### Step one: analyses of the genes targeted by current ADHD drugs

The genes targeted by the current FDA-approved ADHD medications were defined by Gaspar and Breen [34]. We examined these genes individually (gene-based tests) and altogether (gene-set analyses) in MAGMA software [35]. Each gene’s degree of association with a phenotype was calculated based on the individual single nucleotide polymorphisms’ (SNP) association *p*-values from their respective GWA studies. SNPs with chromosomal positions within the boundaries of a gene (start and end of a primary transcript) were assigned to that gene (i.e. the default settings of MAGMA). The 1000 Genomes CEU population was used as the reference panel to correct for linkage disequilibrium (LD). We conducted gene-based analyses of all genes on autosomal chromosomes. Genes represented by a single parameter (i.e. only one association signal) in MAGMA were excluded. To evaluate each gene’s contribution to the examined gene-set, the association *p*-value of each gene was converted to a Z-value and used as an outcome variable for a regression model with gene-set membership as a predictor. Gene size, gene-sets’ gene density and LD were taken into account to adjust for possible confounding effects and prevent spurious association.

#### Step two: analyses of genes and pathways within the druggable genome

The gene associations with ADHD, its co-morbidities and quality of life phenotypes were tested on two levels: (a) DNA variation and (b) gene expression. The first was tested in MAGMA as described above. The latter was tested in S-PrediXcan [36]. In short, S-PrediXcan first predicts tissue-specific gene expression level of each gene based on the reference transcriptome data [37] and then estimates the correlation between that level and a phenotype using GWA summary statistics. Given that ADHD is believed to be a disorder of the central nervous system, we restricted our S-PrediXcan analyses to its tissues. S-PrediXcan analyses were performed using the default settings of the software. Type 2 diabetes was excluded from these analyses as the available summary statistics did not contain the necessary data.

The biological pathways were defined as determined by Gene Ontology (GO) [38] and the Kyoto encyclopaedia of genes and genomes (KEGG) [39]. We restricted our analyses to pathways represented by more than 10 and less than 1000 genes. The analyses were conducted in MAGMA [35] as described above.

### Characterisation of the druggable genome loci associated with ADHD and/or its co-morbidities and quality of life phenotypes

To address our third aim, we explored the pharmacology of genes (or their encoded proteins) pinpointed in our statistical analyses.

To identify the pharmacological agents, we developed a systematic pipeline utilising publically available databases, where the agents were assessed in four stages: (1) FDA-approved drugs, (2) drugs in clinical trials, (3) compounds reported in the Drug–Gene Interaction Database (DGIdb, http://dgidb.org/), and (4) small molecule compounds with reported molarity measurement for bioactivity.

First, we evaluated each gene of interest in Uniprot (https://www.uniprot.org/) and characterised the identified FDA-approved drugs and compounds in clinical trials using Drugbank (https://www.drugbank.ca/), CLUE Repurposing Hub (https://clue.io/repurposing-app), and DGIdb databases. The FDA approval and clinical trial status of the compounds were crosschecked using the publically available FDA labels and ClinicalTrials.gov (https://clinicaltrials.gov/) database. The paediatric approval status was investigated on Medscape (https://reference.medscape.com/) and in FDA labels. For FDA-approved compounds, the approved indication reported in FDA label was noted. For compounds in clinical trials, the indication was researched in ClinicalTrials.gov, applying the following filters: “not yet recruiting”, “recruiting”, “enroling by invitation”, “active, not recruiting” and “completed”, in order to select trials that are currently active. In addition, the mechanism of action on the specific gene of interest was noted from Drugbank.

Next, the genes were researched as targets in ChEMBL (https://www.ebi.ac.uk/chembl/), downloading all compounds reported to interact with a gene of interest along with their reported “Target Associated Bioactivity” as their affinity or potency to the human gene product stated in molarity (referred to as “Bioactivity”). The compounds from ChEMBL were then prioritised from the lowest “Bioactivity” value to the highest and, in order to investigate the most relevant compounds, the top 50 were characterised further by being individually investigated in PubChem (https://pubchem.ncbi.nlm.nih.gov/) to confirm their target-associated bioactivity.

## Results

### Definition of druggable genome and ADHD co-morbidities

The druggable human genome has been estimated to comprise 4479 genes [29], 3826 of which were represented by more than one association signal in the ADHD GWA data. These genes were present in 2758 pathways (2560 GO and 198 KEGG).

### Genetic data

We obtained summary statistics from GWA studies for ADHD, eight neuropsychiatric disorders, three cardiometabolic diseases, five immune diseases and three quality of life phenotypes (Table 1).

### Statistical analyses

In ADHD, we examined 3826 genes and 2759 gene sets in the druggable genome (2560 GO, 198 KEGG and one set of genes targeted by FDA-approved ADHD drugs), bringing the Bonferroni-corrected significance threshold to *p* = 7.59E–06.

In the co-morbidities and quality of life phenotypes, we examined 385 genes and three gene sets (two GO pathways and one set of FDA-approved ADHD genes) in the druggable genome. The Bonferroni-adjusted significance thresholds for these analyses was set to *p* = 1.29E–04.

Associations stronger than the determined Bonferroni thresholds were considered significant.

#### Step one: analyses of the genes targeted by current ADHD drugs

We identified 23 genes targeted by the FDA-approved ADHD drugs (and revealing more than one independent association signal in ADHD GWA summary statistics). Individually, none of these genes showed significant association with ADHD (Table 2). For co-morbid conditions, several significant associations were noted (Table S1). The strongest one was observed between *DRD2* and the frequency of alcohol consumption (*p* = 2.88E–08), followed by associations between the same gene (*DRD2*) and SCZ (*p* = 1.55E–07), *CYP2D6* and SCZ (*p* = 1.81E–06), *CHRM2* and major depressive disorder (*p* = 2.56E–06). In addition, *SLC6A3* revealed significant association with sleep duration (*p* = 2.41E–05). All of these genes encode protein targets of atomoxetine. Furthermore, *SLC6A3* is also targeted by MPH and AMP, while *DRD2* is a secondary target of MPH and AMP.

Examining all the genes as a set revealed no significant association with neither ADHD nor its co-morbidities (Table S2).

#### Step two: analyses of genes and pathways within the druggable genome

##### Analyses of genetic variation (MAGMA)

For ADHD, four loci on chromosomes one, three, four and twelve showed significant association (Table 3). The locus on chromosome one contains seven druggable genes, while the other three loci contain one druggable gene each (Figs. S1–S4). The most significant association was observed on chromosome one (*ST3GAL3* gene, *p* = 3.10E–12, Table 3 and Fig. S1). While the loci on chromosomes one, four and twelve revealed strong association signals at the individual SNP level (Figs. S1–S3), the locus on chromosome three did not (Fig. S4) and, thus, was excluded from further analyses.

**Fig. 2:**
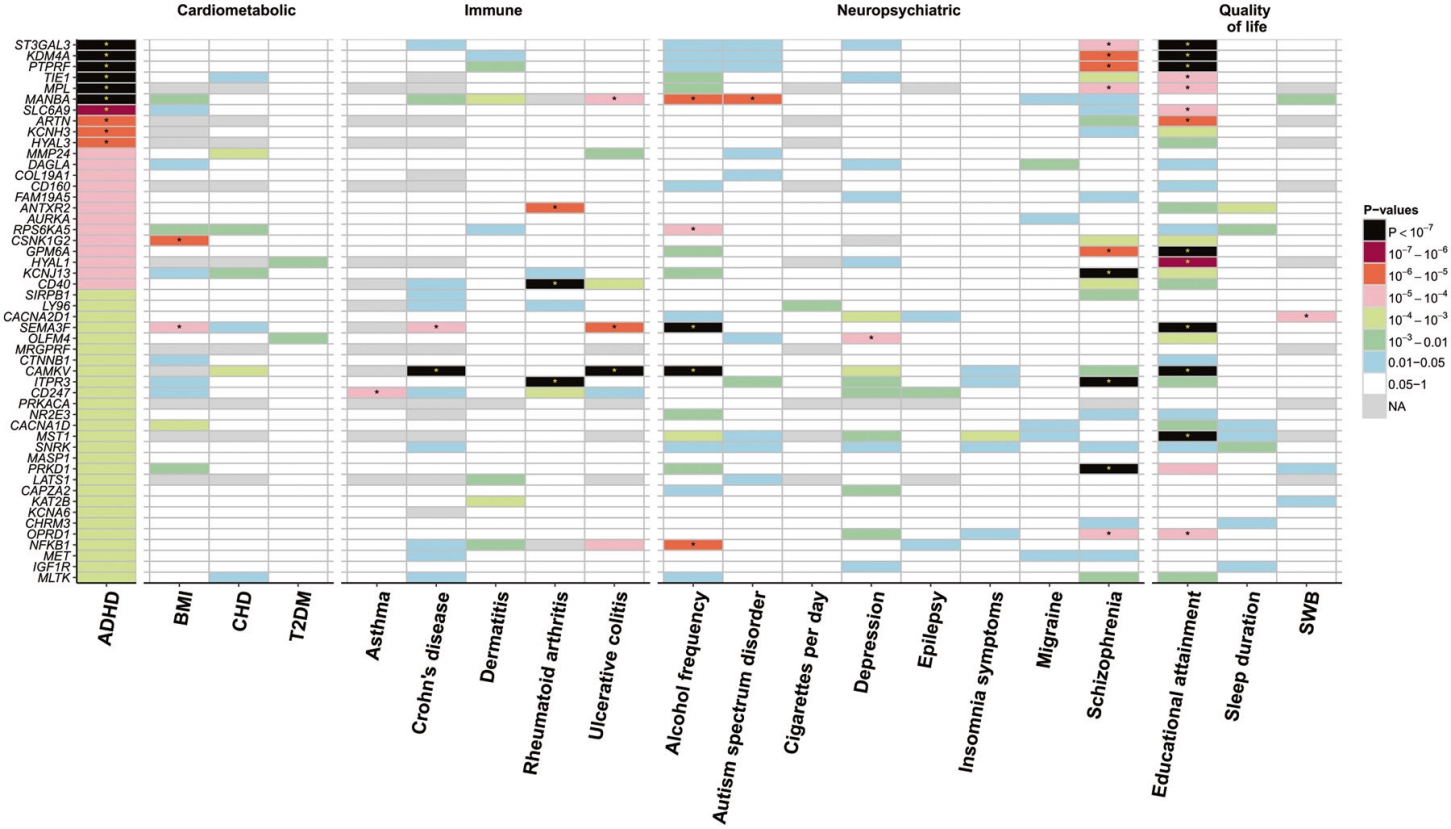
Tile plot of the association between druggable genes and ADHD (*p* < 0.001), its co-morbid conditions of ADHD and quality of life phenotypes. ADHD; attention deficit/hyperactivity disorder, BMI; body mass index, CHD; coronary heart disease, T2DM; type 2 diabetes mellitus, SWB; subjective well-being

For the co-morbidities, the most significant association was noted between *ITPR3* and rheumatoid arthritis (*p* = 4.88E–38, Fig. 2 and Table S3). This gene was also significantly associated with SCZ (*p* = 1.13E–09). Among the three loci associated with ADHD, those on chromosomes one and four revealed druggable genes also significantly associated with SCZ, ulcerative colitis, autism spectrum disorder and the frequency of alcohol consumption (Fig. 2 and Table S3). In addition, educational attainment showed the highest number of significantly associated genes (Table S3).

##### Analyses of gene expression (S-PrediXcan)

In total, we examined 13 tissues of the central nervous system (Fig. S5). Transcriptome data for 2225 druggable genes (also examined in MAGMA) were present in at least one tissue of central nervous system.

For ADHD, the expression levels of two genes—*MANBA* (p = 1.63E–07 in”cerebellar hemisphere”) and *LEPRE1* (*p* = 5.05E–09 in “frontal cortex”)—showed significant association and 18 additional genes showed signs of suggestive association (*p* < 0.001) (Table S4).

For co-morbidities, the expression of 13 genes revealed significant associations with a number of examined phenotypes (Fig. S5 B–S, Table S4). The most significant association was observed between the expression of *HLA-DPB1* and rheumatoid arthritis (*p* = 2.96E–45 in “cerebellum”, Table S4). The expression levels of the two genes that showed significant association with ADHD (*MANBA* and *LEPRE1*) were also significantly associated with body mass index, rheumatoid arthritis and SCZ (Table S4).

Overall, gene expression analyses highlighted five druggable genes significantly associated with ADHD and/or its comorbidities and quality of life phenotypes in addition to those prioritised in analyses of genetic variation.

#### Pathway analyses

For ADHD, no significant association was noted among either GO or KEGG pathways, with the strongest signal observed for negative regulation of protein binding (GO:0032091, *p* = 1.5E–04). Two GO pathways revealed nominal associations with ADHD (*p* < 0.001, Table S5) and were analysed for association with its co-morbidities and quality of life phenotypes, showing no significant associations (Table S5).

The results of KEGG pathways are summarised in Table S6.

### Characterisation of the druggable genome loci associated with ADHD and/or its co-morbidities and quality of life phenotypes

#### Genetic variation loci

Out of the nine druggable genes located within the three loci significantly associated with ADHD, the proteins encoded by five of them are interacting with pharmaceuticals that are FDA-approved or in clinical trials: *PTPRF*, *TIE1*, *MPL*, *SLC6A9* and *KCNH3* (Table 3). Among their indications we noted malignancies, autoimmune diseases, neuropsychiatric disorders (including ADHD, Parkinson’s and Alzheimer’s diseases), metabolic disorder, haematopoietic processes, inflammation, atrial fibrillation and spinal cord injury (Table S7). No FDA-approved drugs or drugs in clinical trials interacted with the druggable locus on chromosome four.

For co-morbid conditions, we examined 14 loci within the druggable genome that all showed suggestive association with ADHD (*p* < 0.001) and significant association with any of the examined co-morbidities. Among the 17 druggable genes within those 14 loci, 13 interact with drugs that are in clinical trials or are FDA-approved, with the majority of indications being autoimmune disorders and/or malignancies (Table S8).

#### Gene expression loci

Among the five genes pinpointed by S-PrediXcan (and not overlapping with those identified in MAGMA), three are targeted by compounds in clinical trials and two of them are also FDA-approved. All of these compounds are nutraceuticals, with malignancies and immune dysfunctions among their indications (Tables S9 and S10).

## Discussion

Despite ADHD being a highly heritable disorder, it has been challenging to utilise genetic information in its treatment. Nonetheless, the more insight we gain into the molecular genetics of ADHD, the more options for its treatment may become available [40]. In this study, we explored the druggable genome in ADHD, its co-morbid conditions and quality of life phenotypes utilising largescale GWA studies. We aimed to address three questions: (1) do any of the genes encoding targets of FDA-approved ADHD drugs show association with ADHD and/or its co-morbidities, (2) are ADHD and/or its co-morbidities and quality of life phenotypes associated with genetic variation and expression within the known druggable genome and if so, (3) can we use those association signals to identify gene targets for novel drug development and/or repurposing to treat ADHD.

To answer the first question, we examined the association between the genes encoding the immediate targets of the first-line ADHD pharmacotherapeutics and ADHD as well as its co-morbidities. We observed no significant association between these genes and ADHD, suggesting that these drugs may act through mechanisms different to those underlying ADHD. However, as the current GWA study on ADHD reveals only a small fraction of the biological processes underlying this condition [30], larger studies are needed to draw any definitive conclusions.

Overall, drugs that are FDA-approved or currently undergoing clinical trials to treat ADHD (e.g. dasotraline) target only a limited number of known pharmacological targets, essentially enhancing catecholamine signalling. This illustrates that all active ADHD drugs belong to a small pharmacological niche and we should aim to move beyond it. Hence, we examined whether any known druggable genes and pathways are associated with ADHD and/or its co-morbidities and quality of life phenotypes (second question), following by pharmacological characterisation of identified associations (third question). These analyses aimed to pinpoint novel avenues for ADHD drug development as well as repurposing. Because the de novo discovery and development of entirely new drugs targeting unique biology of a disorder is a tedious and expensive process with a low success rate, the possibility of repurposing already existing drugs towards new indications may be more effective [40]. Here, we highlight some of these potential new targets, although this list is not comprehensive.

Within the loci associated with ADHD, 5 druggable genes encode proteins interacting with drugs that are FDA-approved or are in clinical trials. The common indications of those pharmaceuticals are autoimmune disorders and malignancies, with some also being tested in clinical trials for treatment of neurodevelopmental disorders. Interestingly, autoimmune disorders and malignancies are also common indications for drugs interacting with genes associated with co-morbidities of ADHD, suggesting that these two fields of research could present novel paths for ADHD treatment.

The locus on chromosome one shows the strongest association with ADHD and also contains the most genes interacting with drugs that are FDA-approved or in clinical trials. Among them, *PTPRF* is the gene with the most prominent association signal. This gene encodes a tyrosine phosphatase, a signalling molecule involved in a myriad of cellular processes, including cell adhesion, neuronal development and functioning [41, 42]. *PTPRF* has mainly been studied in the context of cancer. However, its involvement in hyperactivity [42] and axonal growth [43] has also been reported. Another ADHD-associated gene interacting with drugs that are FDA-approved and/or are in clinical trials is *SLC6A9*, a gene encoding a glycine transporter that is targeted by such compounds as bitopertin, sarcosine and glycine [44]. In ADHD, glycine supplementation is currently under investigation as a potential treatment [45]. Similarly, sarcosine has also been tested as a possible ADHD drug, although the preliminary analyses indicate that its effect may be limited to oppositional symptoms only [46].

Outside the chromosome one locus, the *KCNH3* gene is also interacting with drugs that are FDA-approved or are in clinical trials. This gene encodes a voltage-dependent potassium channel, a selective inhibitor of which was recently described [47]. It is also a non-specific target of blood–brain barrier penetrating drug dalfampridine [48] used to relieve the symptoms of multiple sclerosis and related neurologic disorders [44, 49]. Knocking out *KCNH3* in mice has been reported to enhance cognitive skills, including attention, further supporting a potential role of dalfampridine-like drugs in the treatment of ADHD [50].

The aforementioned druggable genes also showed significant association with educational attainment, suggesting that drugs targeting them may have a possible impact on quality of life of ADHD patients.

The analyses of correlation between ADHD and gene expression levels in brain pinpointed druggable genes *MANBA* and *LEPRE1*, among which only *LEPRE1* interacts with a number of compounds in clinical trials, such as nutraceutical ascorbate, succinic acid and L-proline. This gene encodes an enzyme needed for collagen synthesis and assembly, which has recently been proposed as a novel therapeutic vista for protection and regeneration of neurons [51]. Moreover, two additional genes, the expression of which correlated with the examined co-morbidities and quality of life phenotypes are also targeted by nutraceuticals.

In pathway analyses, the GO pathway of negative regulation of protein binding (GO:0032091) showed the strongest, albeit non-significant, association with ADHD. This pathway encompasses any process that negatively affects any protein binding, such as actin binding (e.g. synaptic plasticity), microtubule binding, receptor binding and homodimerization activity of a protein. The latter processes affect a G-protein-coupled receptor signalling, tapping into the largest class of targets in current drug development [52, 53] and presenting a myriad of potential opportunities for new drug discoveries in ADHD. Indeed, one of the novel approaches to pharmacotherapy of ADHD is the use of fasoracetam that acts on G-protein coupled glutamate receptor [40].

Among the examined co-morbidities, the neuropsychiatric (mostly SCZ) and immune groups revealed significant associations, with 12 genes interacting with compounds that are FDA-approved or in clinical trials. Interestingly, one of these genes is *KCNJ13*, encoding a druggable potassium channel targeted by dalfampridine, the same compound that also targets the KCNH3-protein discussed above.

The gene that revealed significant associations with the largest number of co-morbidities is *SEMA3F*. This gene also showed significant association with educational attainment. *SEMA3F* encodes semaphoring-3F protein involved in cell signalling, affecting cell adhesion and migration and being explored mostly in cancer therapies [54, 55]. Nonetheless, the range of therapeutic potential of semaphorins is large [56].

Our study has some limitations. As we examined associations observed in GWA studies, where it is difficult to obtain adequate sample sizes to detect associations of small effects, our findings are limited by their statistical power. Moreover, as we imposed a sample size limit of 20,000 individuals, some of the co-morbid conditions, where GWA studies of such size were not available, were replaced by proxy phenotypes.

The current statistical methods allow us to identify chromosomal loci only. Further studies on the genes of interest as well as fine mapping are needed to unambiguously establish which gene(s) lies on the causal pathway to developing ADHD. This knowledge would allow for a higher resolution search for therapeutic targets, especially on chromosome one locus where the LD structure is particularly complicated.

The gene expression analyses have several limitations [57], including the confounding by genetic associations due to LD, implying a possible substantial bias towards genes located in the loci revealing genome-wide association with the examined trait. In addition, the available transcriptome data are limited and are not available in many relevant tissues (e.g. lack of expression data for *KCNH3* in brain tissues in reference transcriptome), preventing a comprehensive investigation of the transcriptome.

As we used publicly available databases, it was not possible to control their quality. Furthermore, the information provided in the utilised drug target databases may be incomplete.

To conclude, we present a framework for assessment of the druggable genome in a disorder, exemplified by ADHD. We present possibilities for drug repurposing (e.g. dalfampridine) and highlight processes of signal transduction and cell adhesion (negative regulation of protein binding, *PTPRF*, *SEMA3F*, *KCNH3*, *KCNJ13*) as potential novel avenues for ADHD treatment. Our findings add to the knowledge on known ADHD drugs and present an exploration of druggable genome associated with ADHD, which may offer intervention at the aetiological level of the disorder.

## Supplementary Material

suppl

## Figures and Tables

**Table 1:** Overview of the examined data of ADHD, its co-morbidities and quality of life phenotypes

Condition	Sample size^b^	Data source and reference	Reason for inclusion (of co-morbidities and quality of life phenotypes)
ADHD	53,293	Psychiatric genetics consortium [30] http://www.med.unc.edu/pgc/results-and-downloads	
**Cardiometabolic co-morbidities**			
Body mass index^c^	322,154	Genetic Investigation of Anthropometric Traits consortium [58] https://portals.broadinstitute.org/collaboration/giant/index.php/GIANT_consortium_data_files	[30, 32]^a^
Coronary heart disease	84,264	Coronary Artery Disease Genome wide Replication and Meta-analysis plus The Coronary Artery Disease Genetics consortium [59] http://www.cardiogramplusc4d.org/data-downloads/	[30]
Type 2 diabetes mellitus	44,414	Diabetes Genetics Replication And Meta-analysis [60] http://diagram-consortium.org/downloads.html	[30]
**Immune co-morbidities**			
Asthma	26,475	GABRIEL Consortium [61] http://www.cng.fr/gabriel/results.html	[31,32]^a^
Crohn’s disease	49,970	International Inflammatory Bowel Disease Genetics Consortium [62] https://www.ibdgenetics.org/downloads.html	[63]
Dermatitis	40,835	Early Genetics and Lifecourse Epidemiology eczema consortium [64] https://data.bris.ac.uk/data/dataset/28uchsdpmubll8uex26ylacqm	[31]
Rheumatoid arthritis	58,284	Meta-analysis of GWA studies on rheumatoid arthritis [65] http://plaza.umin.ac.jp/yokada/datasource/software.htm	[30]
Ulcerative colitis	43,823	International Inflammatory Bowel Disease Genetics Consortium [62] https://www.ibdgenetics.org/downloads.html	[63, 66]
**Neuropsychiatric co-morbidities**			
Alcohol intake frequency^d^	336,965	UK biobank [67] https://docs.google.com/spreadsheets/d/lb3oGI21Ut57BcuHttWaZotQcI0-mBRPyZihz87Ms_No/edit#gid=1209628142t	[31]
Autism spectrum disorder	46,351	Psychiatric genetics consortium [68] http://www.med.unc.edu/pgc/results-and-downloads	[69, 70]
Number of Cigarettes smoked in a day^d^	38,181	Tobacco and Genetics Consortium [71] https://www.med.unc.edu/pgc/results-and-downloads	[30, 31]
Epilepsy	34,852	International League Against Epilepsy Consortium on Complex Epilepsies [72] http://www.epigad.org/page/show/gwas_index	[73]
Insomnia symptoms^e^	113,006	UK Biobank / Hammerchlag et al. [33] http://ctg.cncr.nl/software/summary_statistics	[32]^a^
Major depressive disorder	260,929	Psychiatric genetics consortium http://www.med.unc.edu/pgc/results-and-downloads [74]	[30, 31,74]
Migraine	205,094	International Headache Genetics Consortium [75]	[31, 32]^a^
Schizophrenia	77,096	Psychiatric genetics consortium [76] http://www.med.unc.edu/pgc/results-and-downloads	[31]
**Quality of life phenotypes**			
Educational attainment	328,917	Social Science Genetic Association Consortium [77] https://www.thessgac.org/data	[30]
Sleep duration	111,980	International Sleep Genetic Epidemiology Consortium [78] https://sleepgenetics.org/downloads/	[32]^a^
Subjective Well Being	298,420	Social Science Genetic Association Consortium [79] https://www.thessgac.org/data	[30]

*ADHD* attention deficit hyperactivity disorder

a Only those co-morbidities were included in this study that revealed well-established evidence for association [32]

b Maximum total sample size

c Proxy for obesity

d Proxy for substance abuse

e Proxy for insomnia disorder

**Table 2:** Association between ADHD and genes targeted by FDA-approved ADHD drugs

Target gene						FDA approved ADHD drug				
						Stimulants		Non-stimulants		
Gene	Chr	Start	End	Number of SNPs	*p*-value	Methylphenidate and derivatives	Amphetamine and derivatives	Atomoxetine	Clonidine	Guanfacine
ADRA1A	8	26605667	26724790	469	0.668	N	Y	N	Y	N
ADRA1B	5	159343790	159399551	118	0.82	N	Y	N	Y	N
ADRA1D	20	4201329	4229721	89	0.499	N	N	N	Y	N
ADRA2A	10	112836790	112840658	5	0.43	Y	Y	Y	Y	Y
ADRA2B	2	96778707	96781984	5	0.399	Y	N	N	Y	Y
CARTPT	5	71014990	71016875	2	0.376	N	Y	N	N	N
CHRM1	11	62676151	62689279	25	0.254	N	N	Y	N	N
**CHRM2**	**7**	**136553416**	**136705002**	**399**	**0.049**	N	N	Y	N	N
CYP2D6	22	42522501	42526908	29	0.689	N	N	Y	N	N
DRD2	11	113280318	113346413	167	0.237	N	N	Y	N	N
HRH1	3	11178779	11305243	308	0.505	N	N	Y	N	N
HTR1B	6	78171948	78173490	5	0.053	N	N	Y	N	N
HTR1D	1	23516993	23521222	9	0.301	N	N	Y	N	N
HTR2A	13	47405685	47471169	207	0.775	N	N	Y	N	N
**HTR6**	**1**	**19991780**	**20006055**	**27**	**0.026**	N	N	Y	N	N
HTR7	10	92500578	92617671	305	0.382	N	N	Y	N	N
NISCH	3	52489134	52527087	68	0.145	N	N	N	Y	N
**NPY1R**	**4**	**164245113**	**164265984**	**48**	**0.032**	N	N	Y	N	N
0PRM1	6	154331631	154568001	609	0.217	N	Y	N	N	N
SLC18A2	10	119000604	119038941	92	0.347	N	Y	N	N	N
SLC6A2	16	55689516	55740104	154	0.223	Y	Y	Y	N	Y
SLC6A3	5	1392909	1445545	100	0.836	Y	Y	Y	N	Y
SLC6A4	17	28521337	28563020	65	0.483	Y	N	Y	N	Y
TAAR1	6	132966123	132967142	3	0.767	N	Y	N	N	N

Genes reaching nominal association *p*-value below 0.05 are highlighted in bold.

“Y” (meaning “yes”) indicates that a gene is targeted by the drug, while “N” (meaning “no”) indicates that a gene is not targeted by the drug. “Chr” refers to the number of a chromosome where the gene of interest is located. “Start” and “End” refer to base pair location of genes of interest.

**Table 3:** The druggable genes located within the three loci significantly associated with ADHD

Locus	Gene	Number of SNPs per gene	*p*-value	Small molecule compound	Biotherapeutic	ADME	Drug in clinical trial	FDA approved drug
Chr1	ST3GAL3	483	3.06E–12	Yes	No	No	No	No
	KDM4A	71	2.11E–11	Yes	No	No	No	No
	PTPRF	225	5.06E–10	Yes	No	No	Yes	Yes
	TIE1	30	2.01E–08	Yes	No	No	Yes	Yes
	MPL	12	4.69E–08	Yes	Yes	No	Yes	Yes
	SLC6A9	67	2.39E–07	Yes	No	No	Yes	Yes
	ARTN	10	3.27E–06	No	No	No	No	No
Chr4	MANBA	203	5.99E–08	Yes	No	No	No	No
Chr12	KCNH3	40	4.23E–06	Yes	No	No	Yes	Yes

“*p*-value” column indicates the strength of association between a gene and ADHD

ADME: genes involved in the absorption, distribution, metabolism, and excretion of drugs; FDA: United States of America Food and Drug Administration; SNP: single nucleotide polymorphism
