# Supplementary figures and images for: Druggable genome in attention deficit/hyperactivity disorder and its co-morbid conditions. New avenues for treatment
Source: Mol Psychiatry. 2019 Oct 18;26(8):4004–15. doi: 10.1038/s41380-019-0540-z (PMC7165040; doi:10.1038/s41380-019-0540-z)

# Chromosome 1 Locus

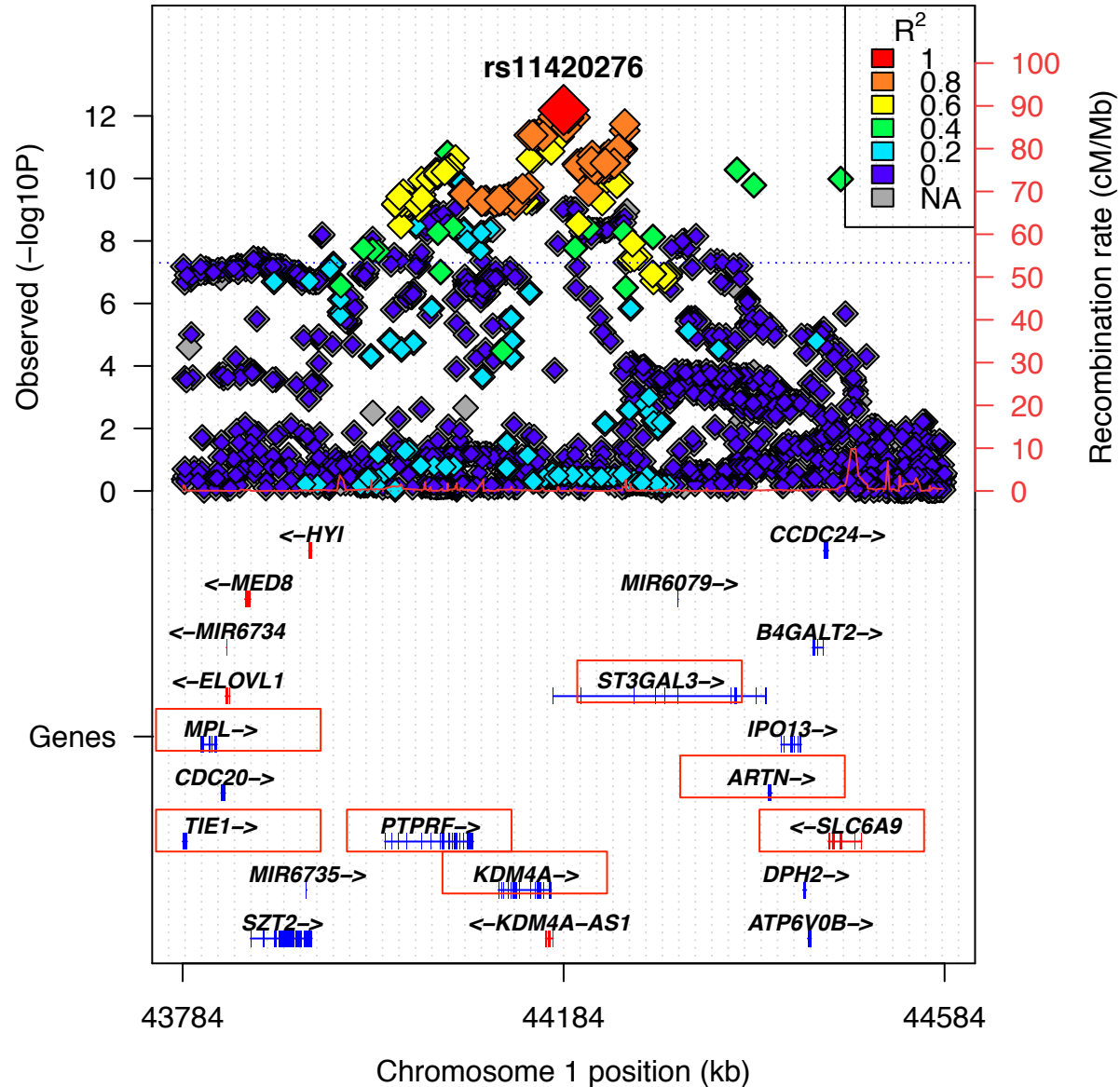

\*the genes in druggable genome are highlighted in red square

Supplement: Supplementary file 1 — Supplementary Figure 1 [file 41380_2019_540_MOESM1_ESM.pdf]

# Chromosome 4 Locus

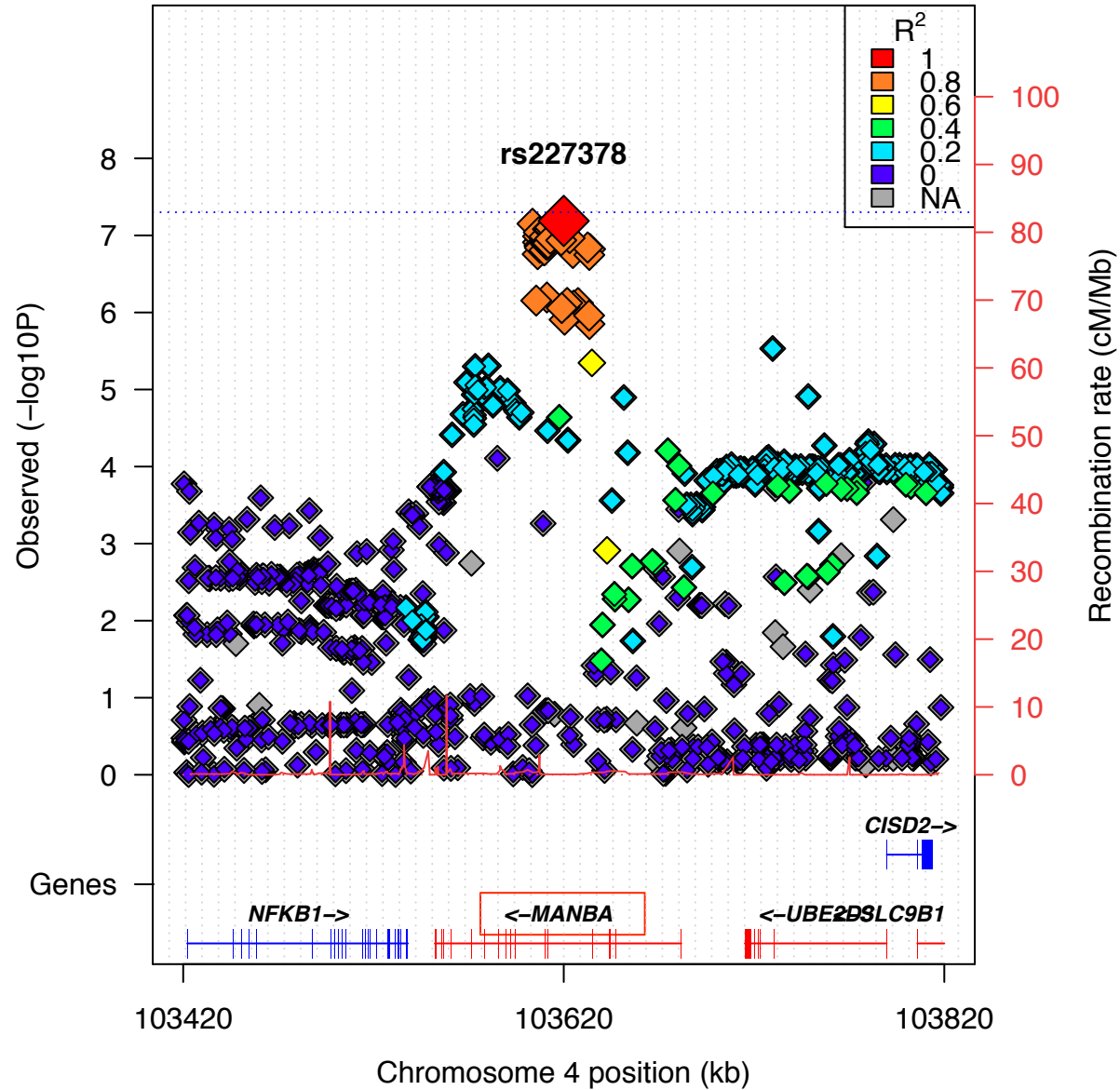

\*the gene in druggable genome is highlighted in red square

Supplement: Supplementary file 2 — Supplementary Figure 2 [file 41380_2019_540_MOESM2_ESM.pdf]

# Chromosome 12 Locus

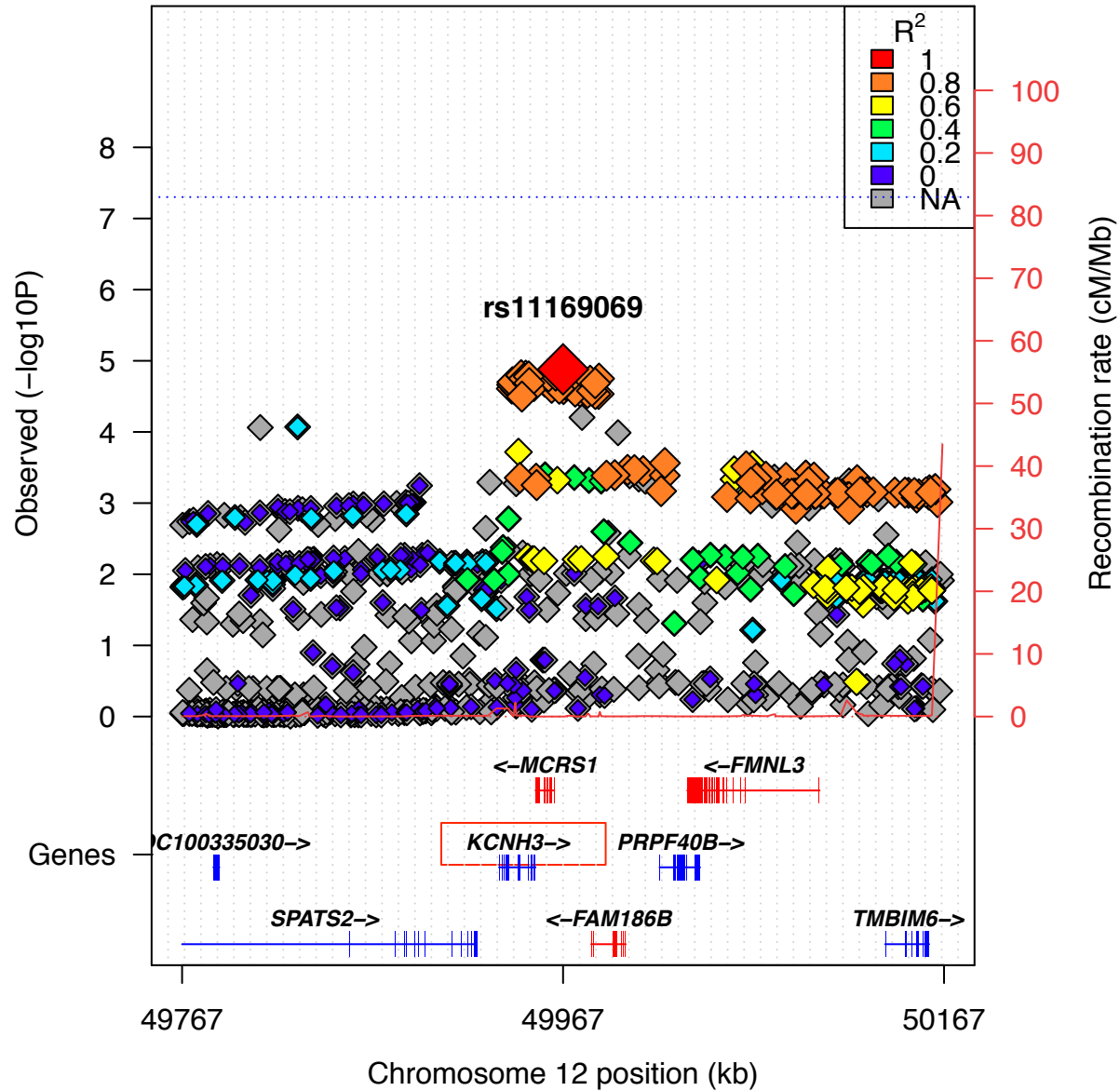

\*the gene in druggable genome is highlighted in red square

Supplement: Supplementary file 3 — Supplementary Figure 3 [file 41380_2019_540_MOESM3_ESM.pdf]

# Chromosome 3 Locus

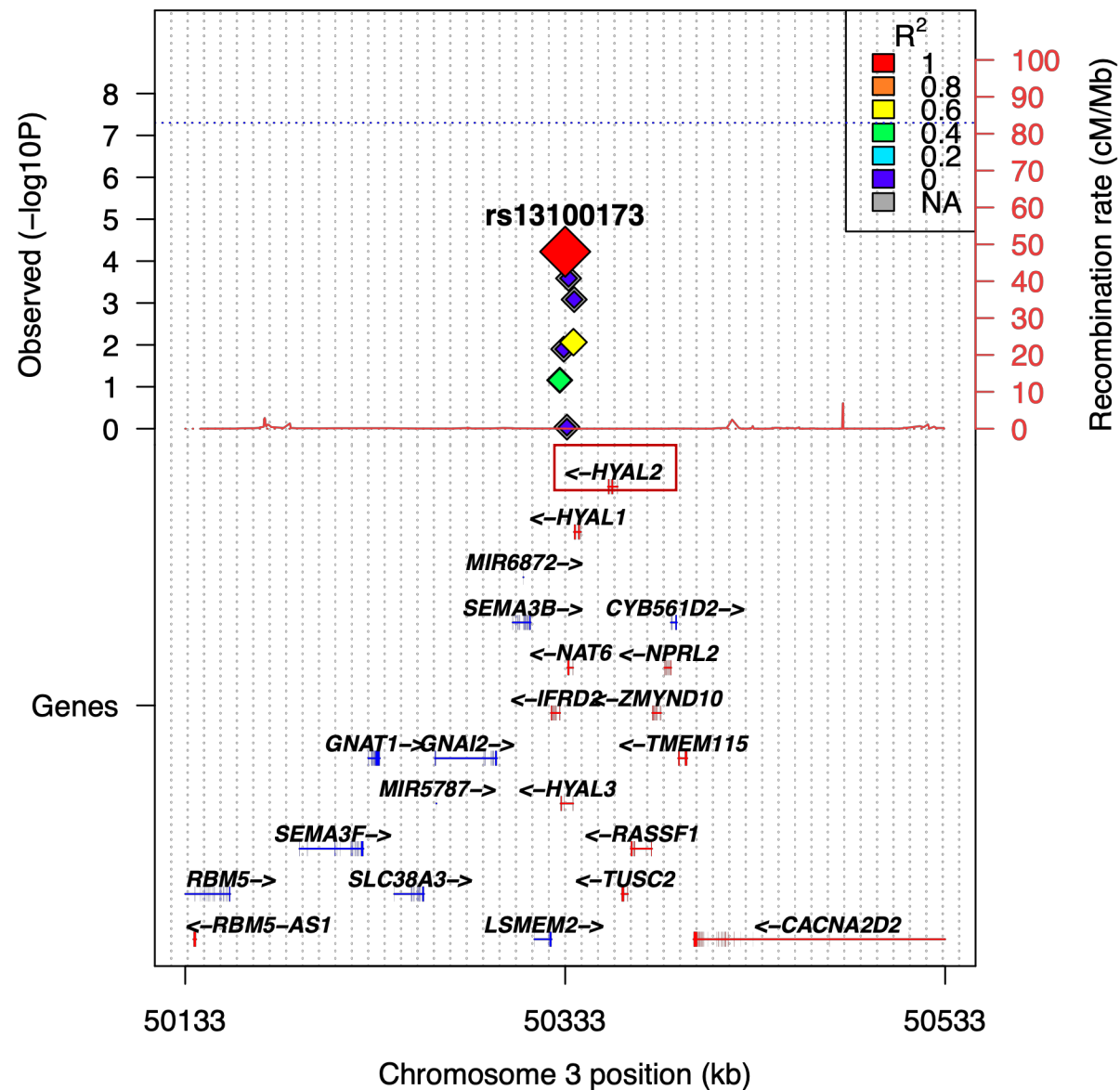

\* the genes in druggable genome are highlighted in red square

Supplement: Supplementary file 4 — Supplementary Figure 4 [file 41380_2019_540_MOESM4_ESM.pdf]
