# Supplementary material for: Druggable genome in attention deficit/hyperactivity disorder and its co-morbid conditions. New avenues for treatment
Source: Mol Psychiatry. 2019 Oct 18;26(8):4004–15. doi: 10.1038/s41380-019-0540-z (PMC7165040; doi:10.1038/s41380-019-0540-z)

Supplementary Figure 5

A) ADHD

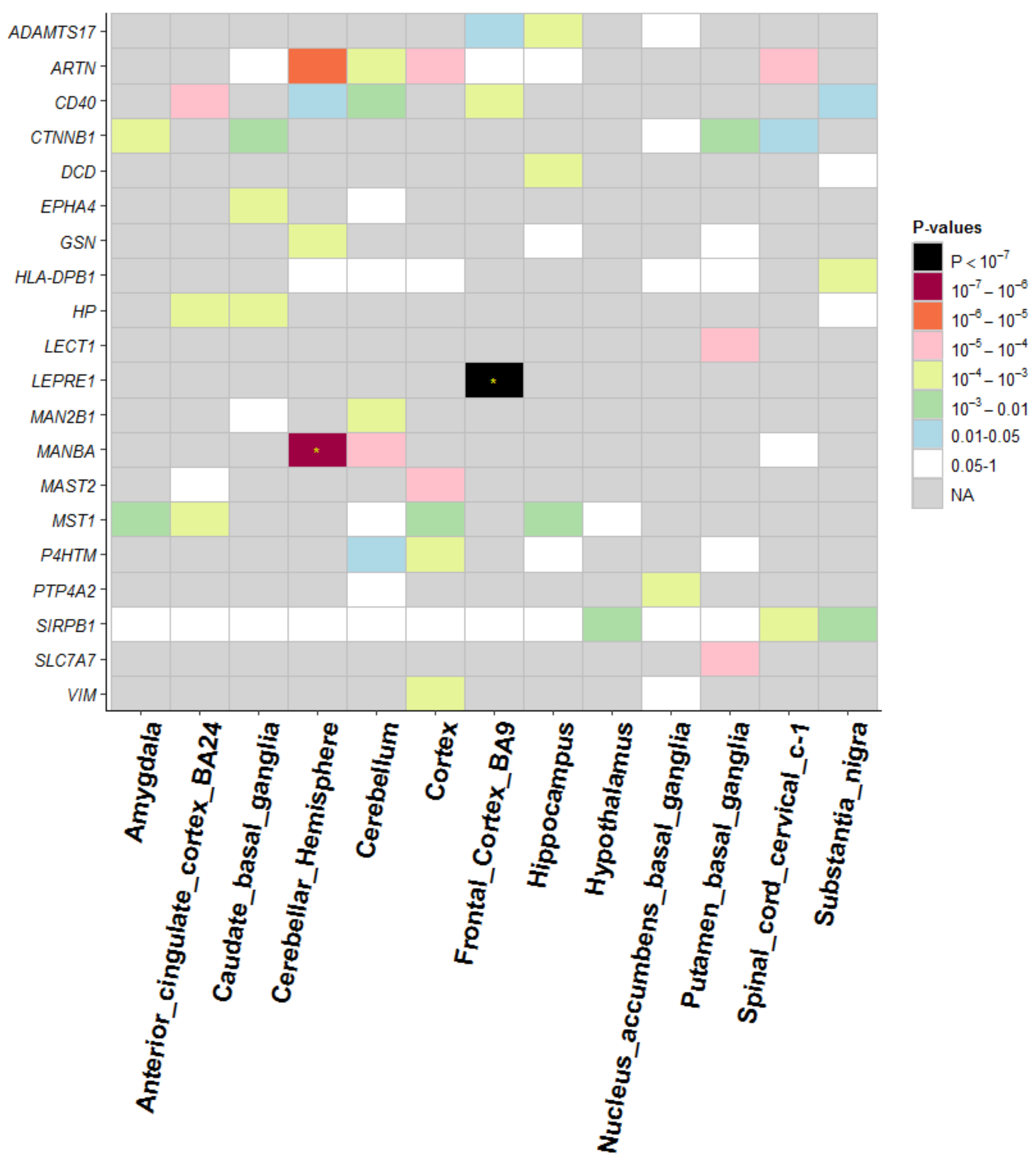

Cardiometabolic

B) Body mass index

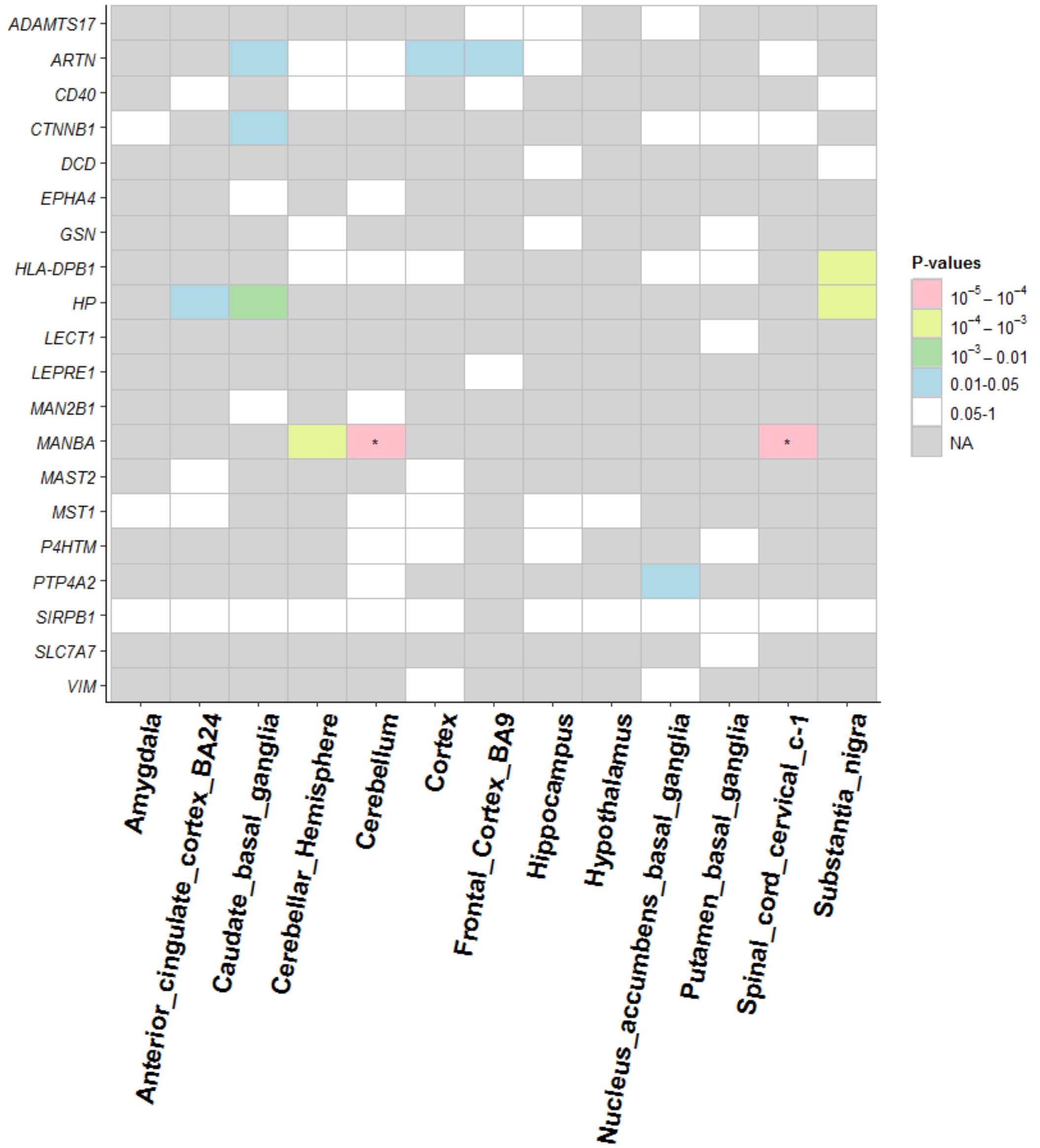

C) Coronary heart disease

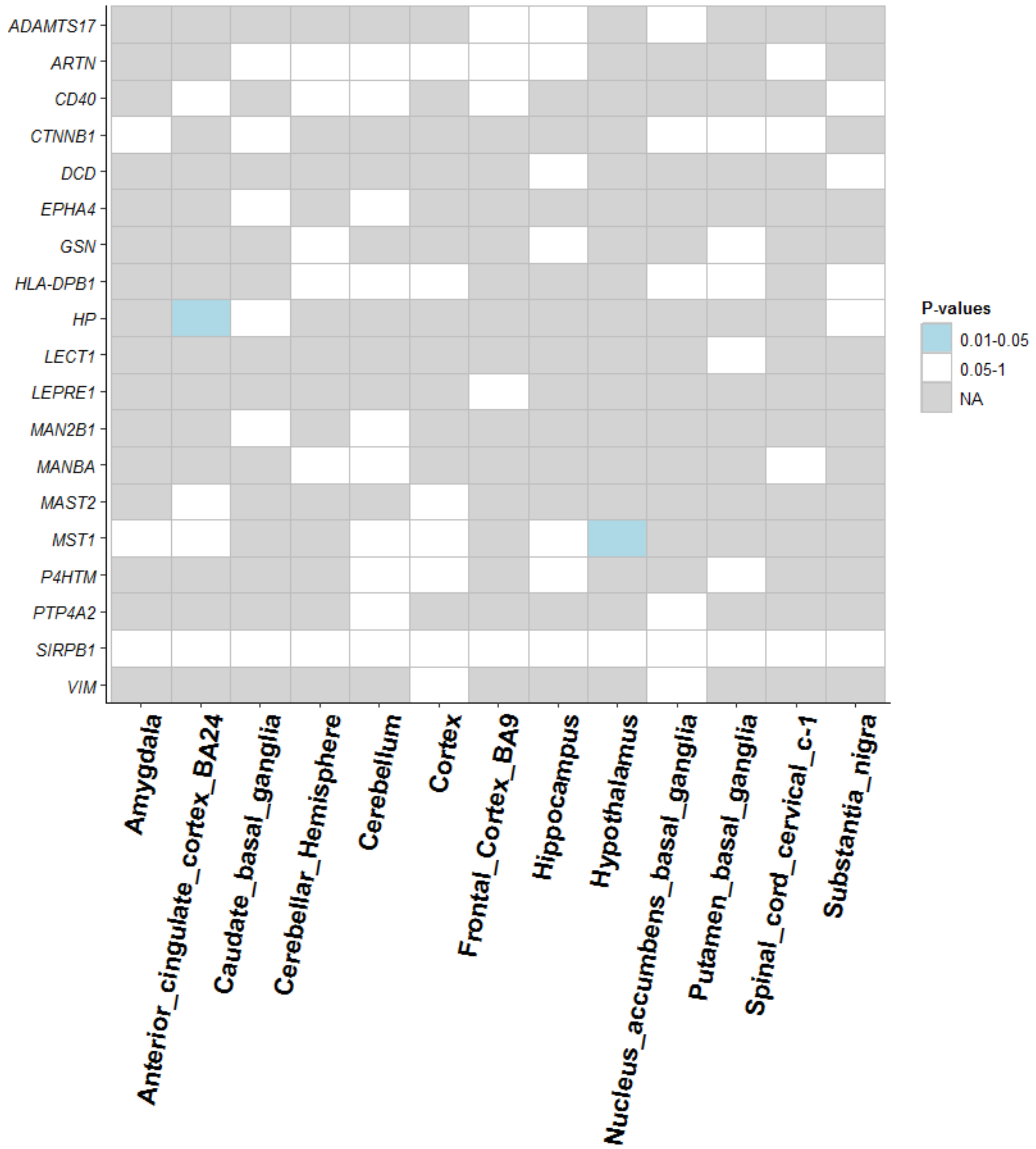

Immune

D) Asthma

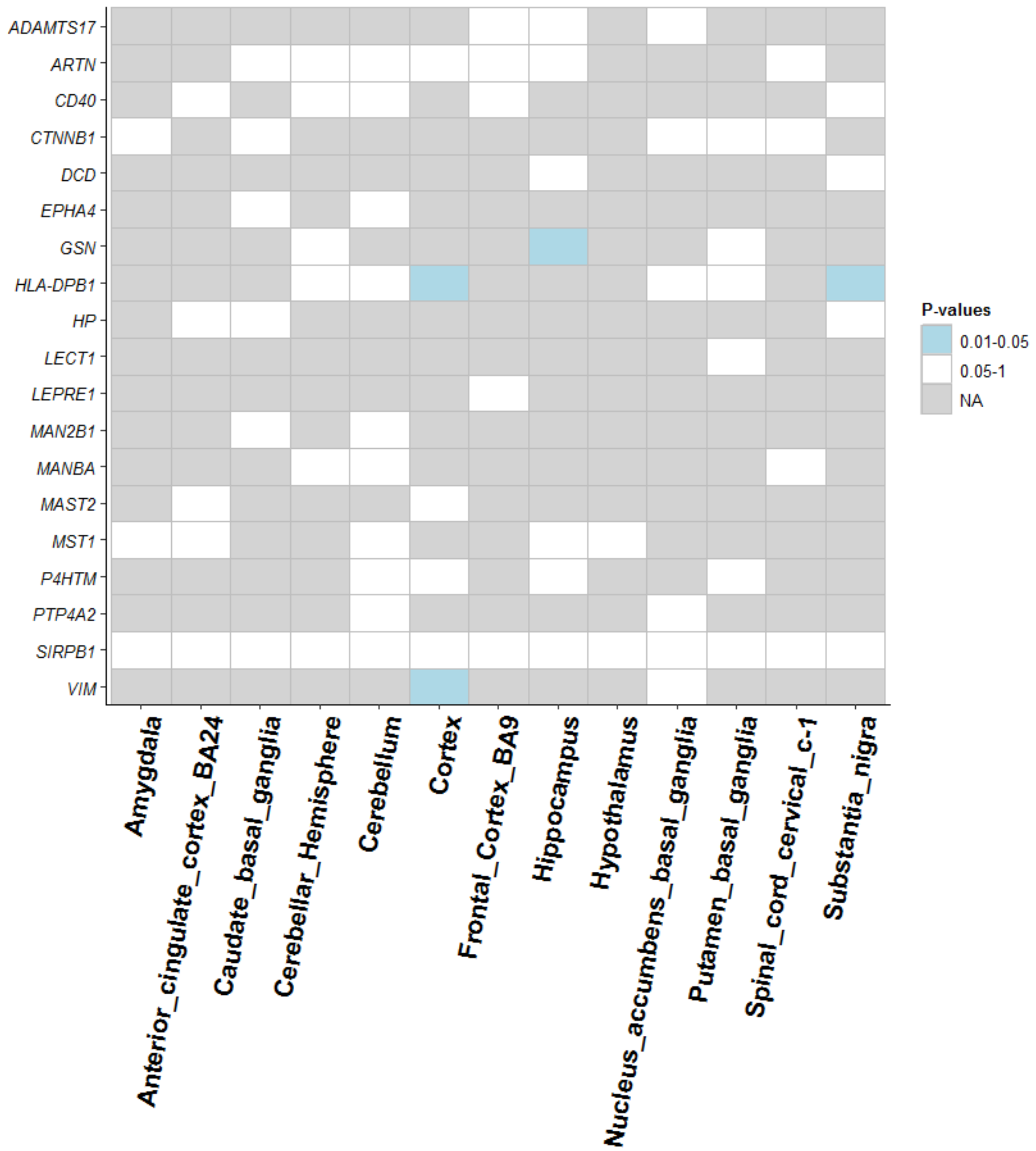

E) Crohn's disease

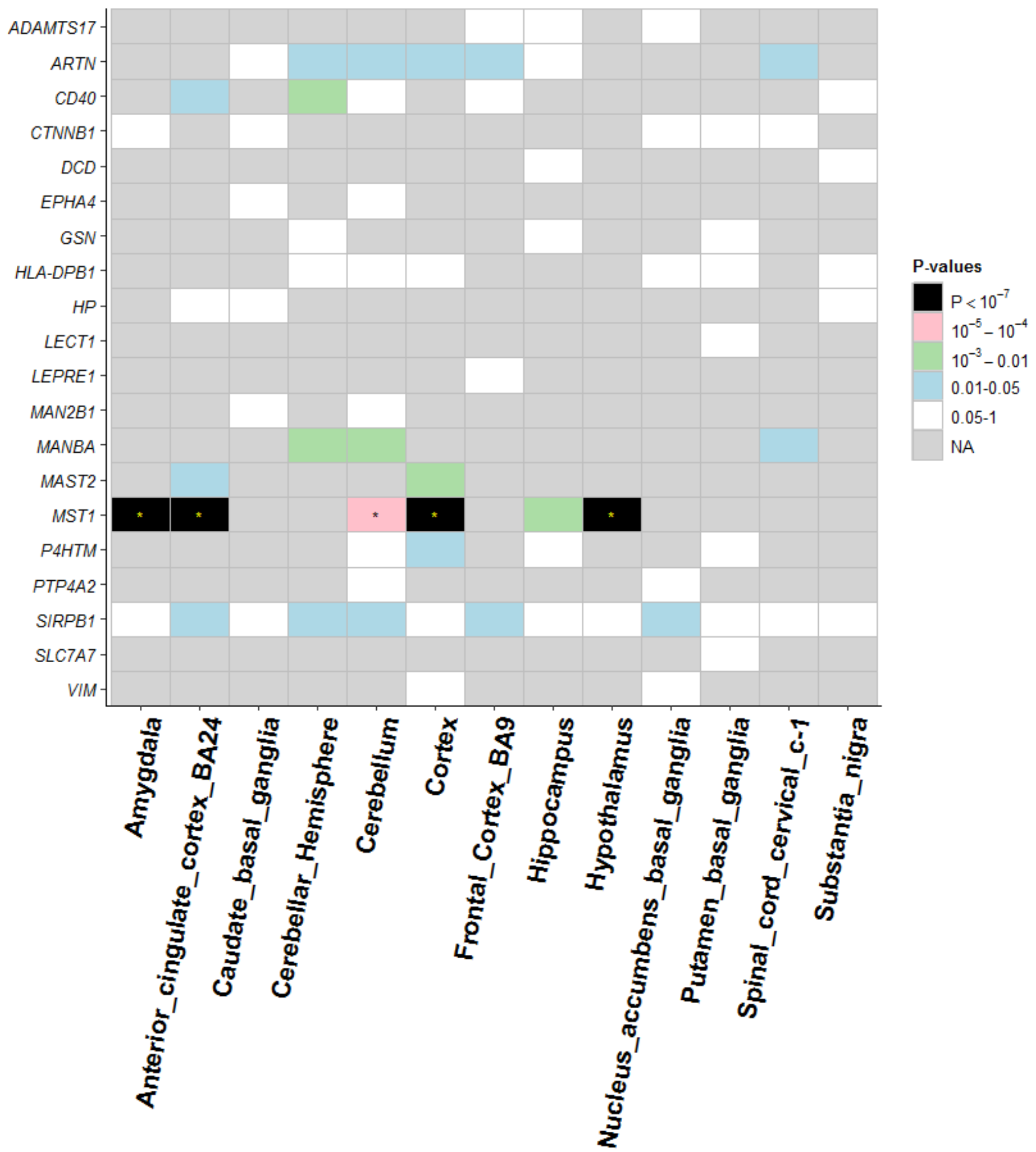

F) Dermatitis

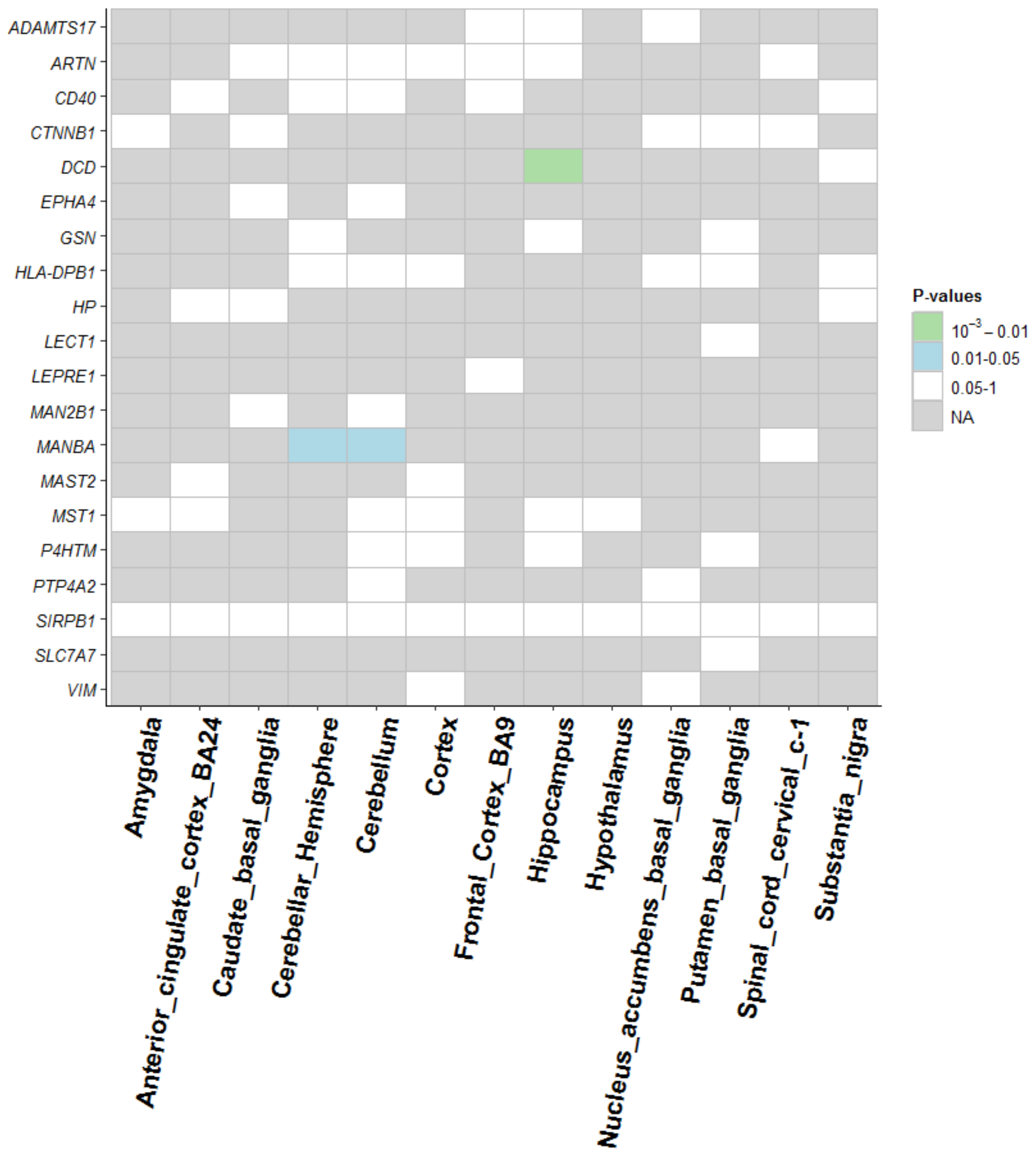

G) Rheumatoid arthritis

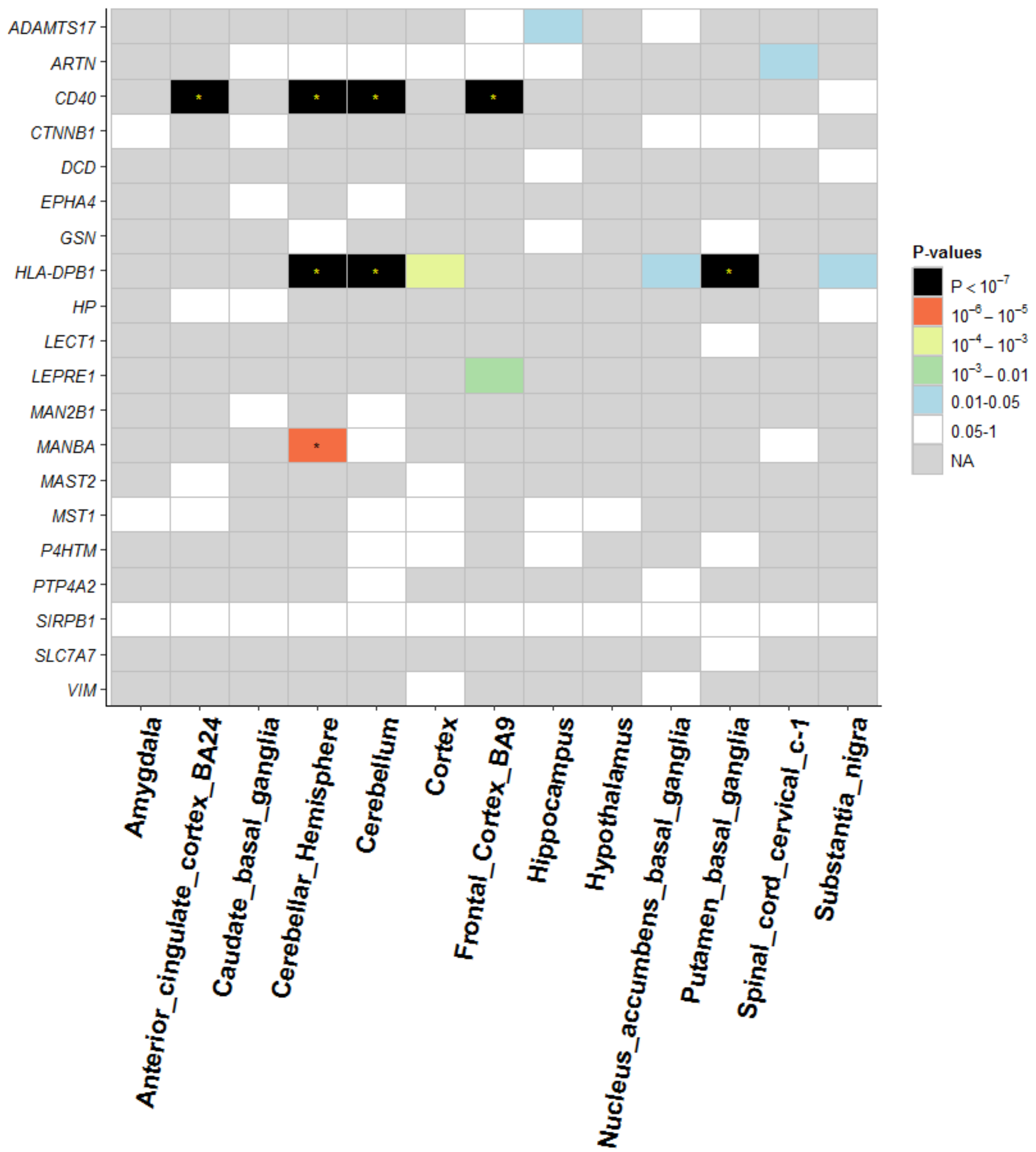

H) Ulcerative colitis

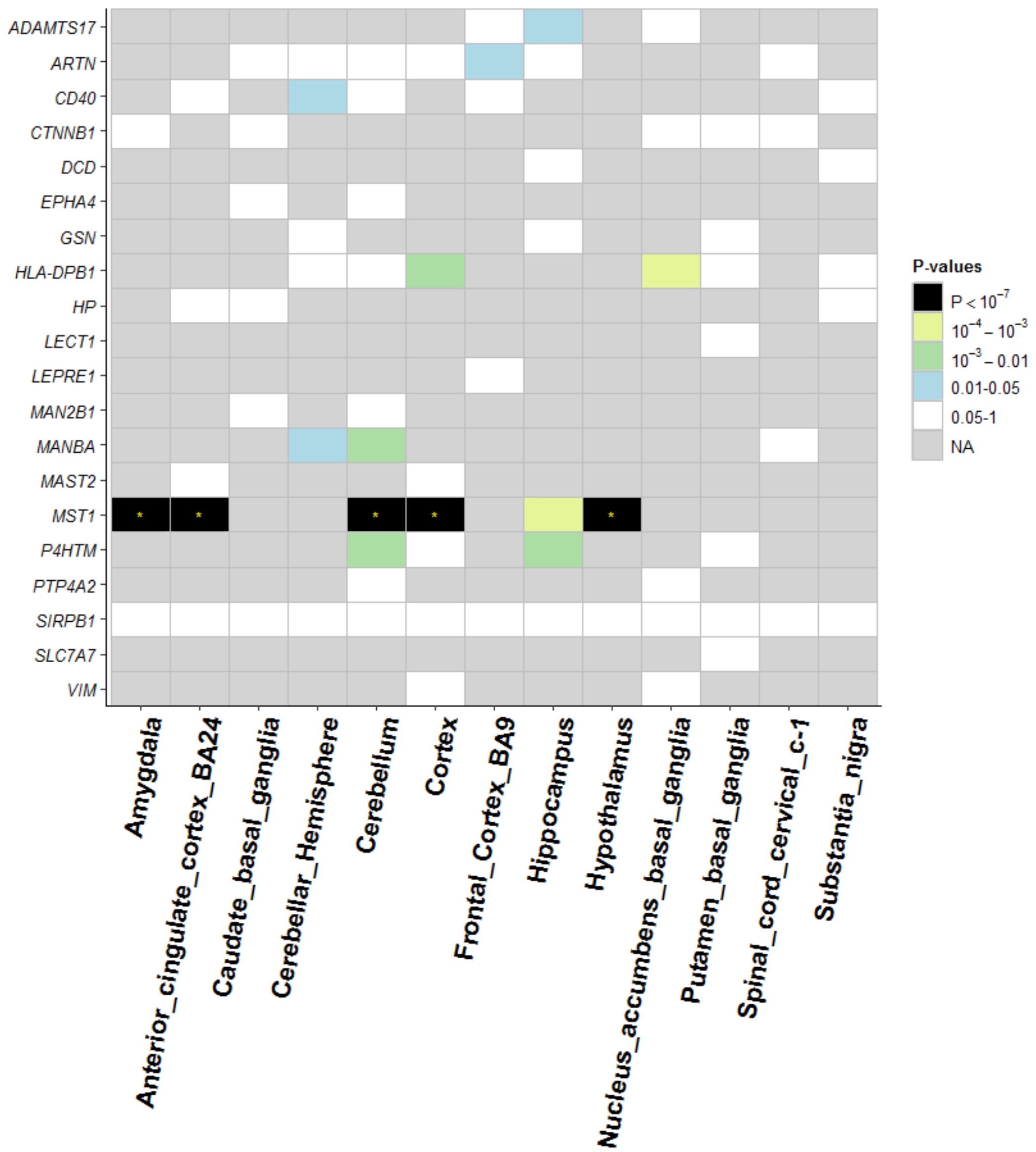

Neuropsychiatric

I) Alcohol frequency

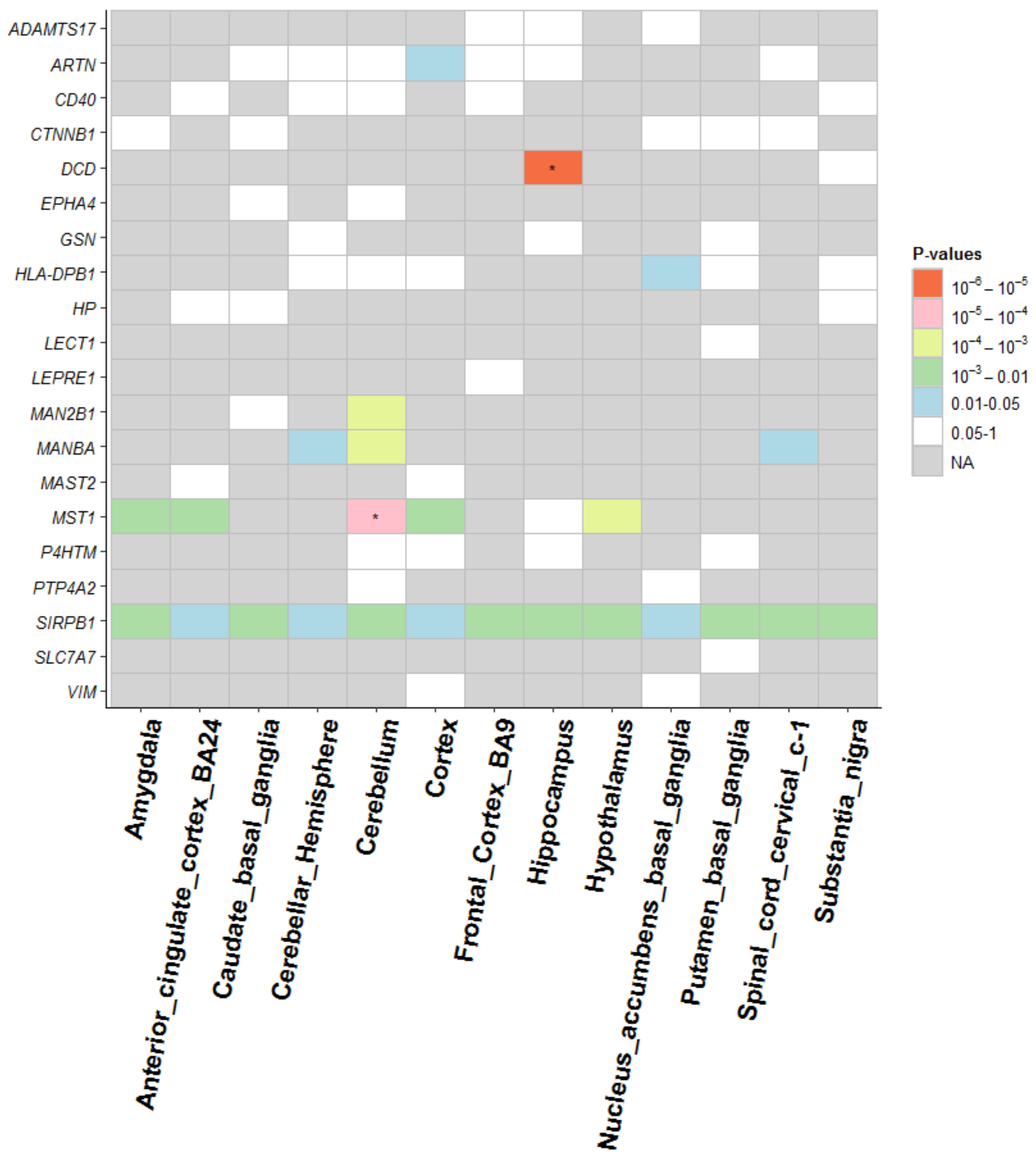

J) Autism spectrum disorder

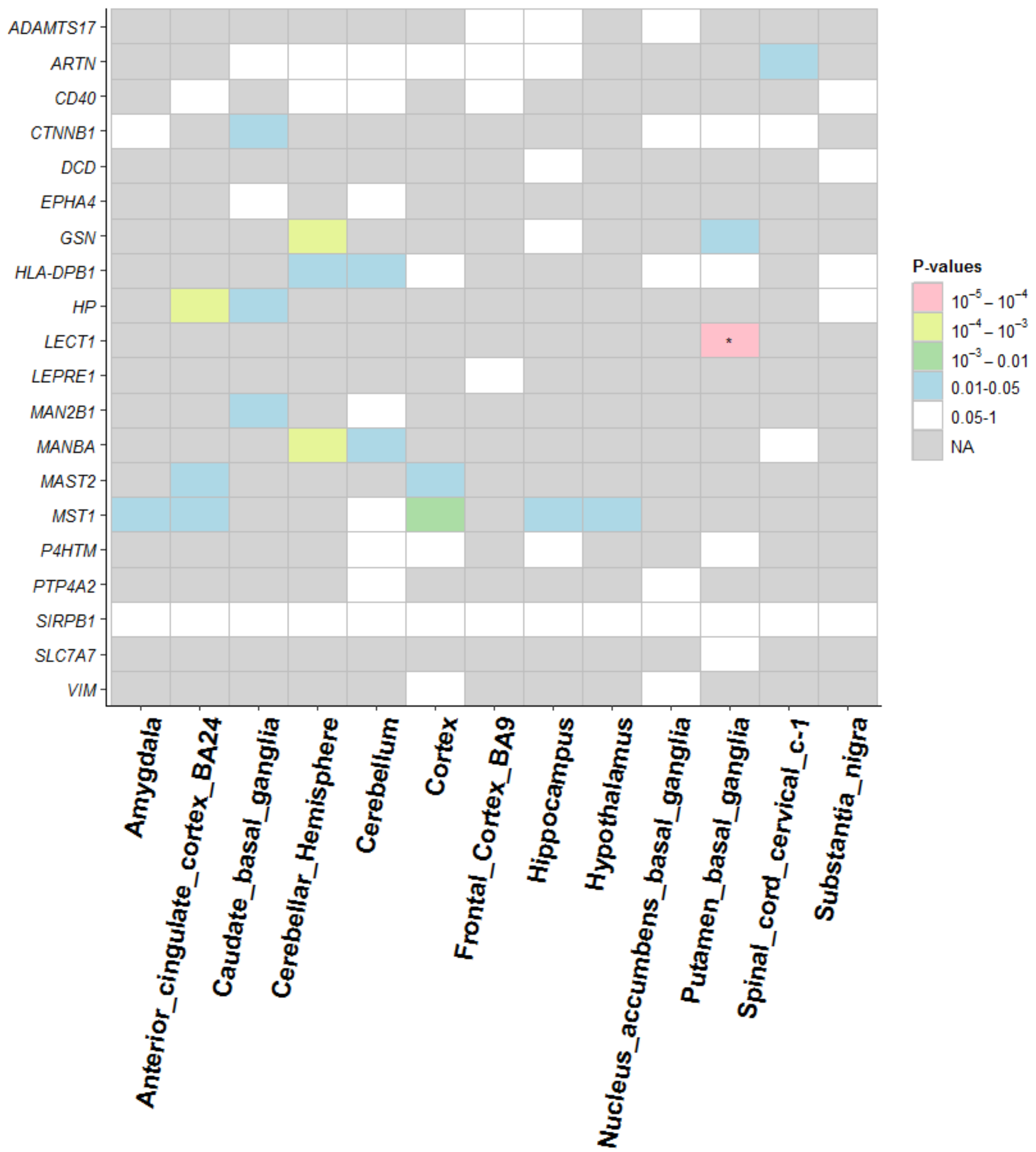

K) Cigarettes per day

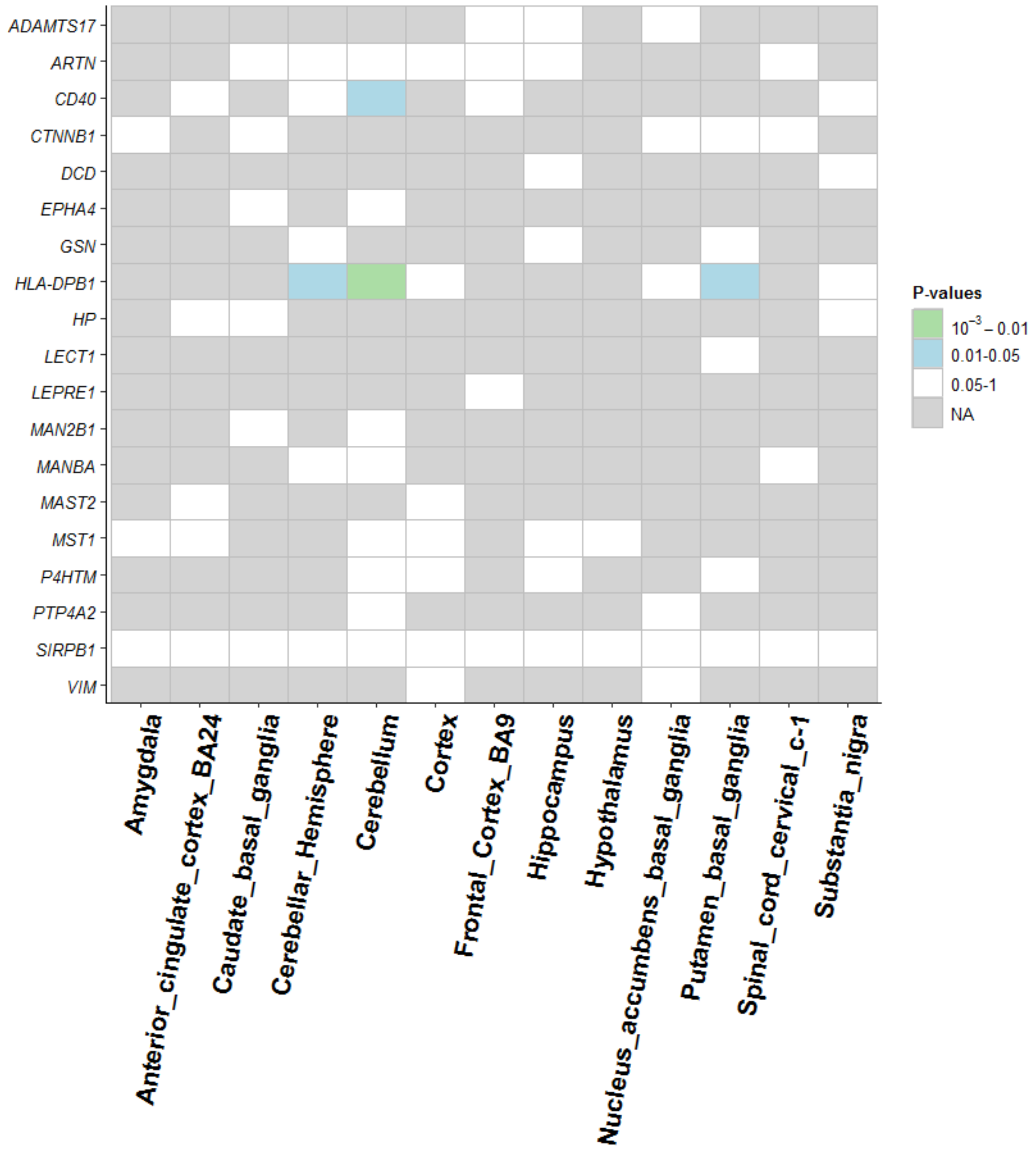

L) Depression

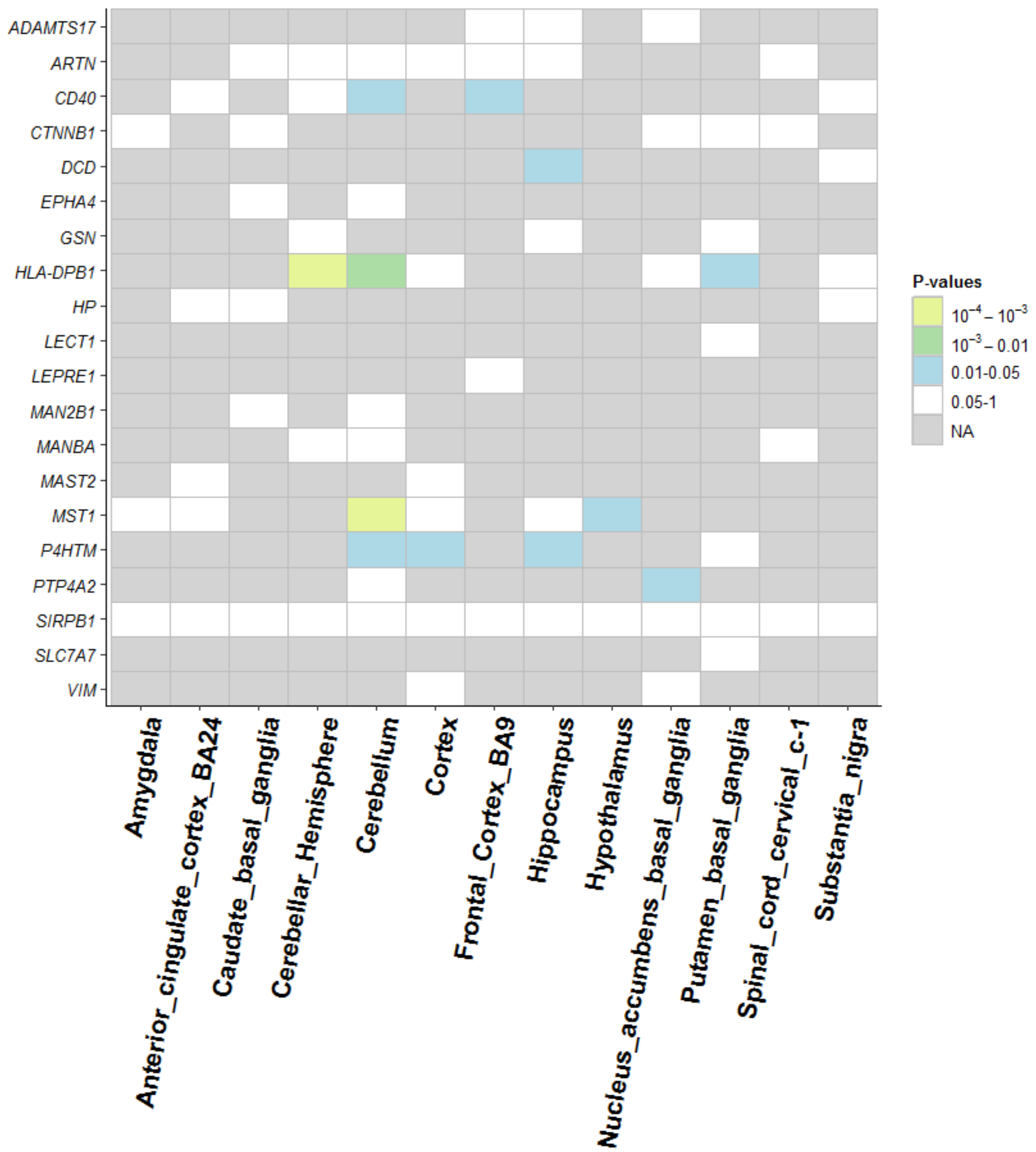

M) Epilepsy

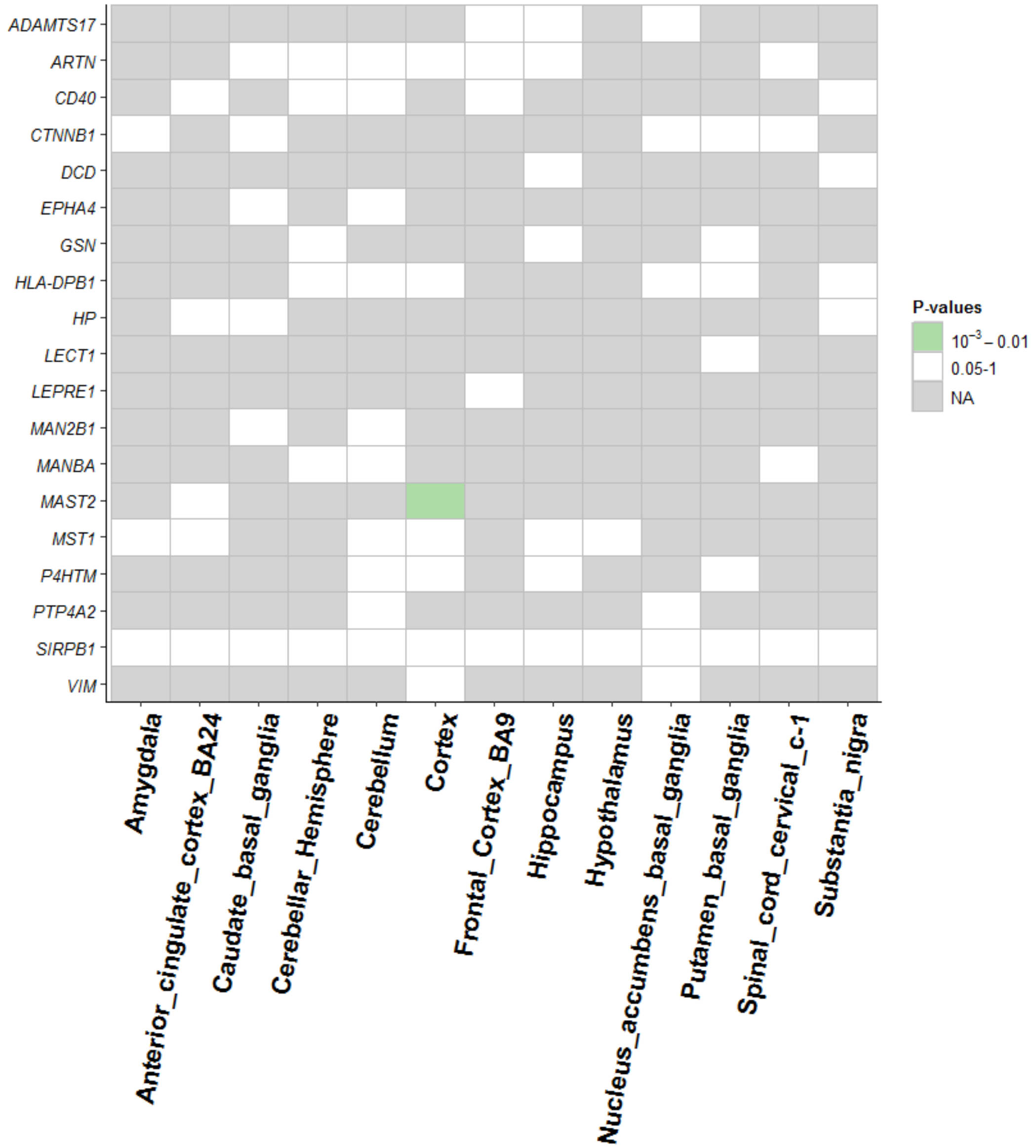

ADAMTS17
ARTN
CD40
CTNNB1
DCD
EPHA4
GSN
HLA-DPB1
HP
LECT1
LEPRE1
MAN2B1
MANBA
MAST2
MST1
P4HTM
PTP4A2
SIRPB1
VIM
Amygdala
Anterior_cingulate_cortex_BA24
Caudate_basal_ganglia
Cerebellar_Hemisphere
Cerebellum
Cortex
Frontal_Cortex_BA9
Hippocampus
Hypothalamus
Nucleus_accumbens_basal_ganglia
Putamen_basal_ganglia
Spinal_cord_cervical_c-1
Substantia_nigra
P-values
10⁻³ – 0.01
0.05-1
NA

N) Insomnia symptoms

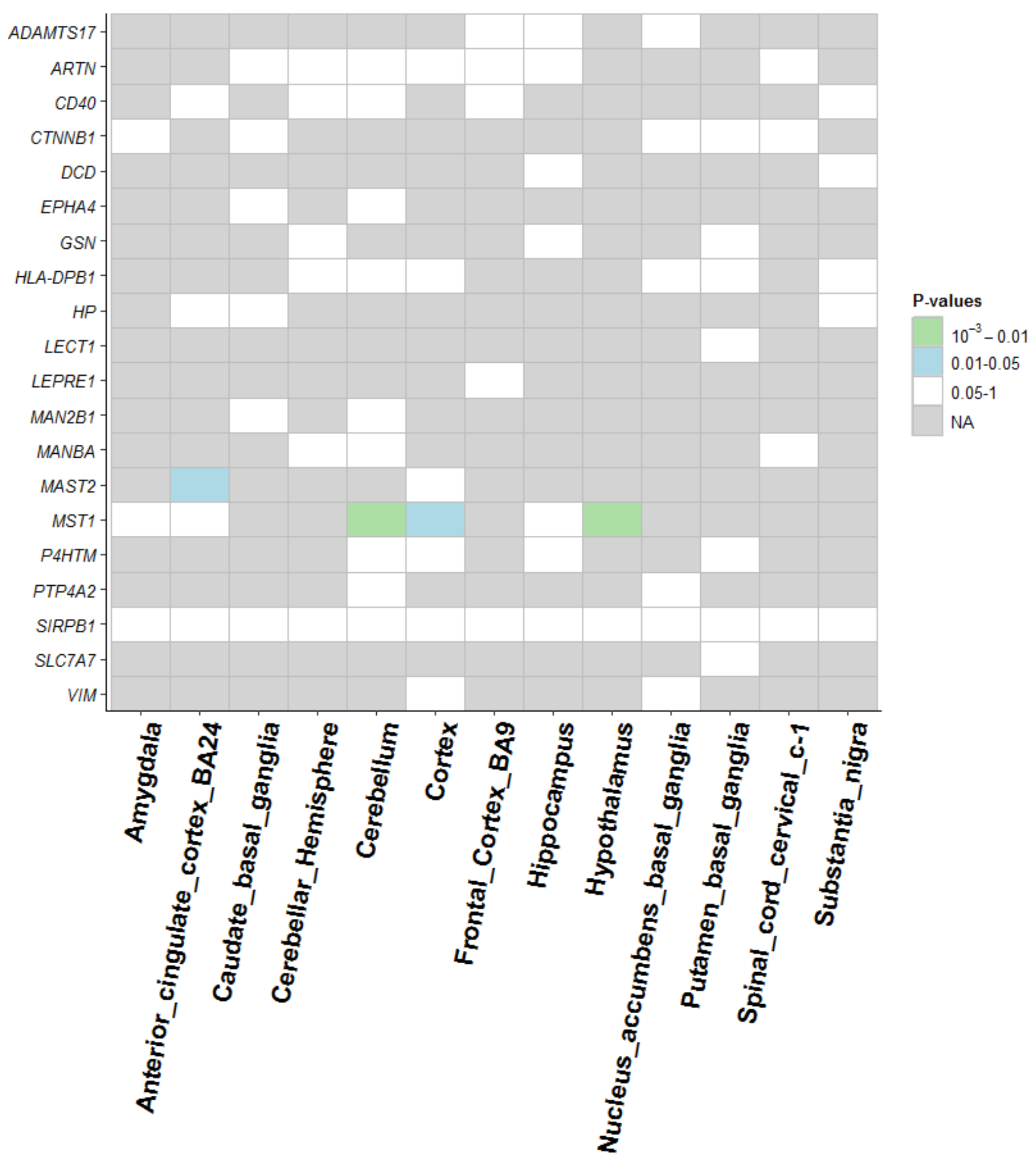

O) Migraine

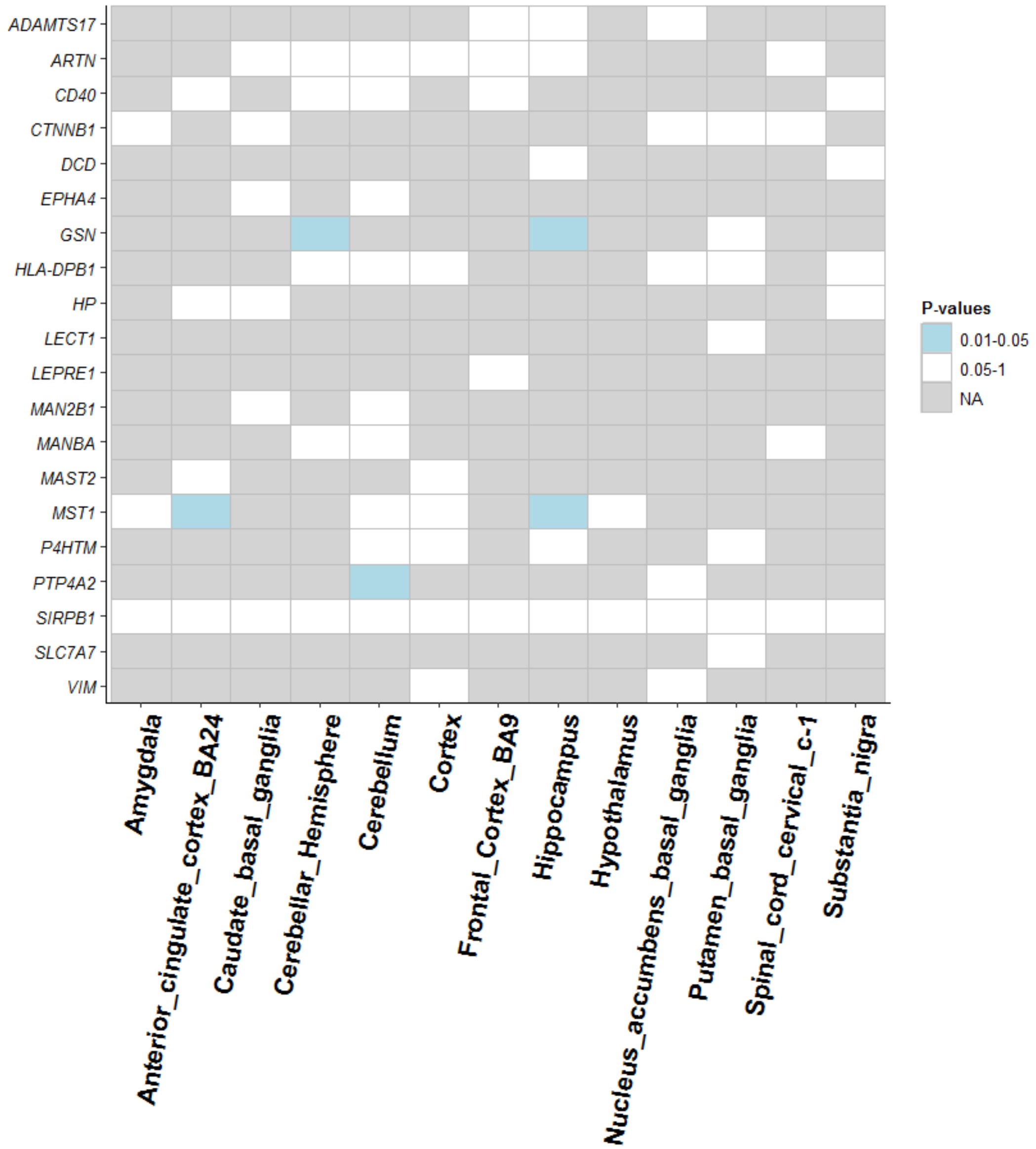
ADAMTS17
ARTN
CD40
CTNNB1
DCD
EPHA4
GSN
HLA-DPB1
HP
LECT1
LEPRE1
MAN2B1
MANBA
MAST2
MST1
P4HTM
PTP4A2
SIRPB1
SLC7A7
VIM
Amygdala
Anterior_cingulate_cortex_BA24
Caudate_basal_ganglia
Cerebellar_Hemisphere
Cerebellum
Cortex
Frontal_Cortex_BA9
Hippocampus
Hypothalamus
Nucleus_accumbens_basal_ganglia
Putamen_basal_ganglia
Spinal_cord_cervical_c-1
Substantia_nigra
P-values
0.01-0.05
0.05-1
NA

P) Schizophrenia

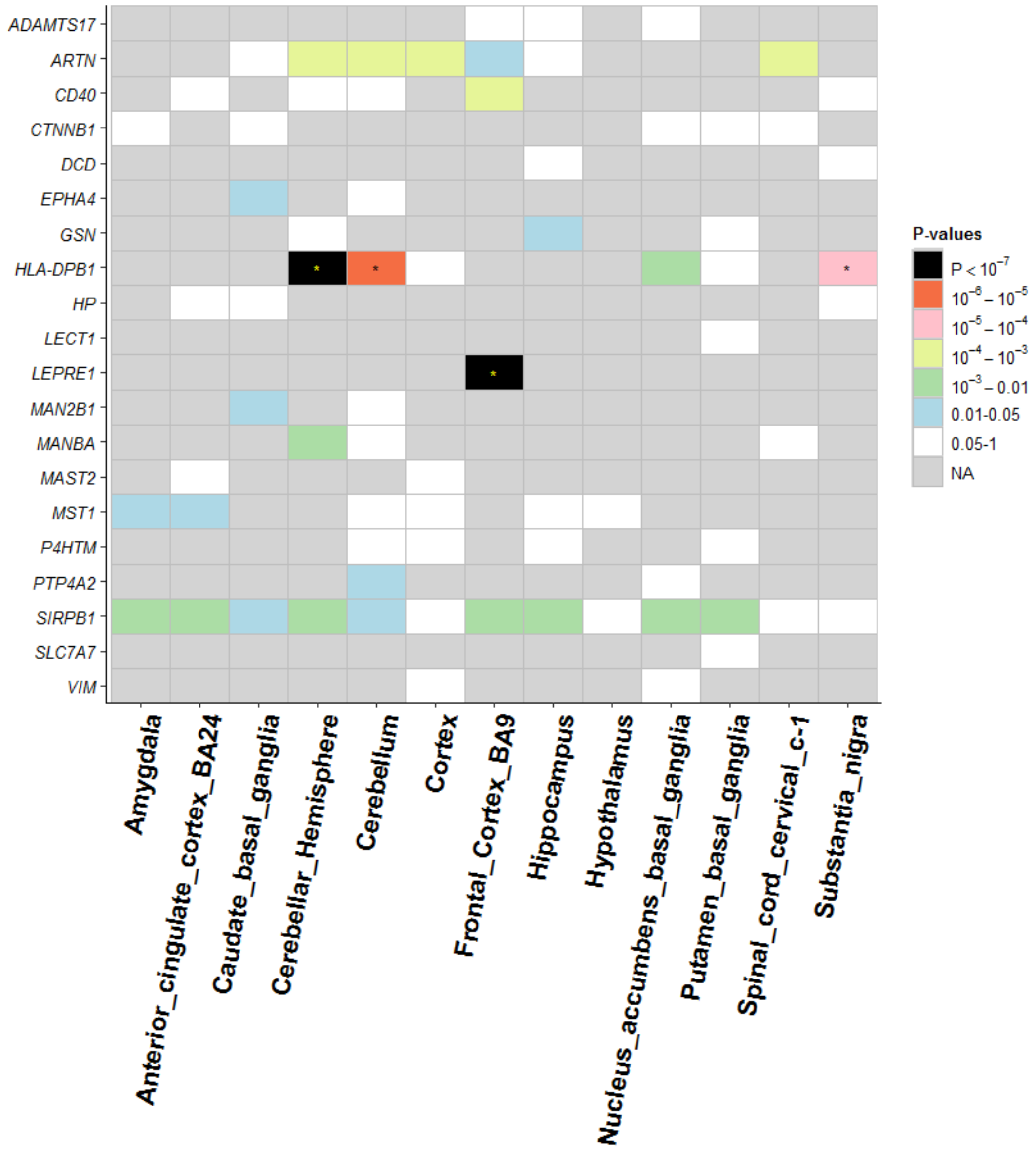

Quality of life

Q) Educational attainment

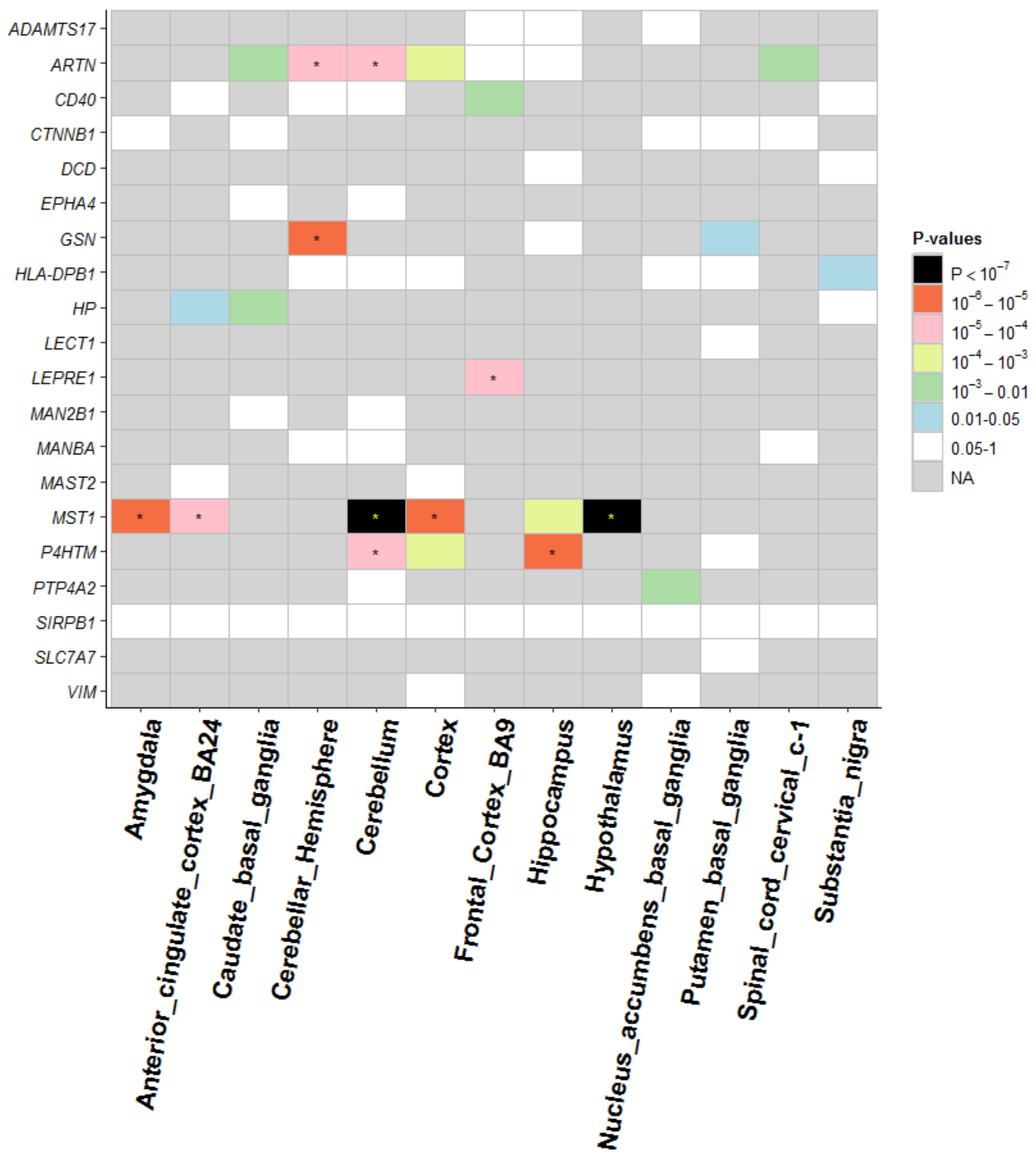

R) Sleep duration

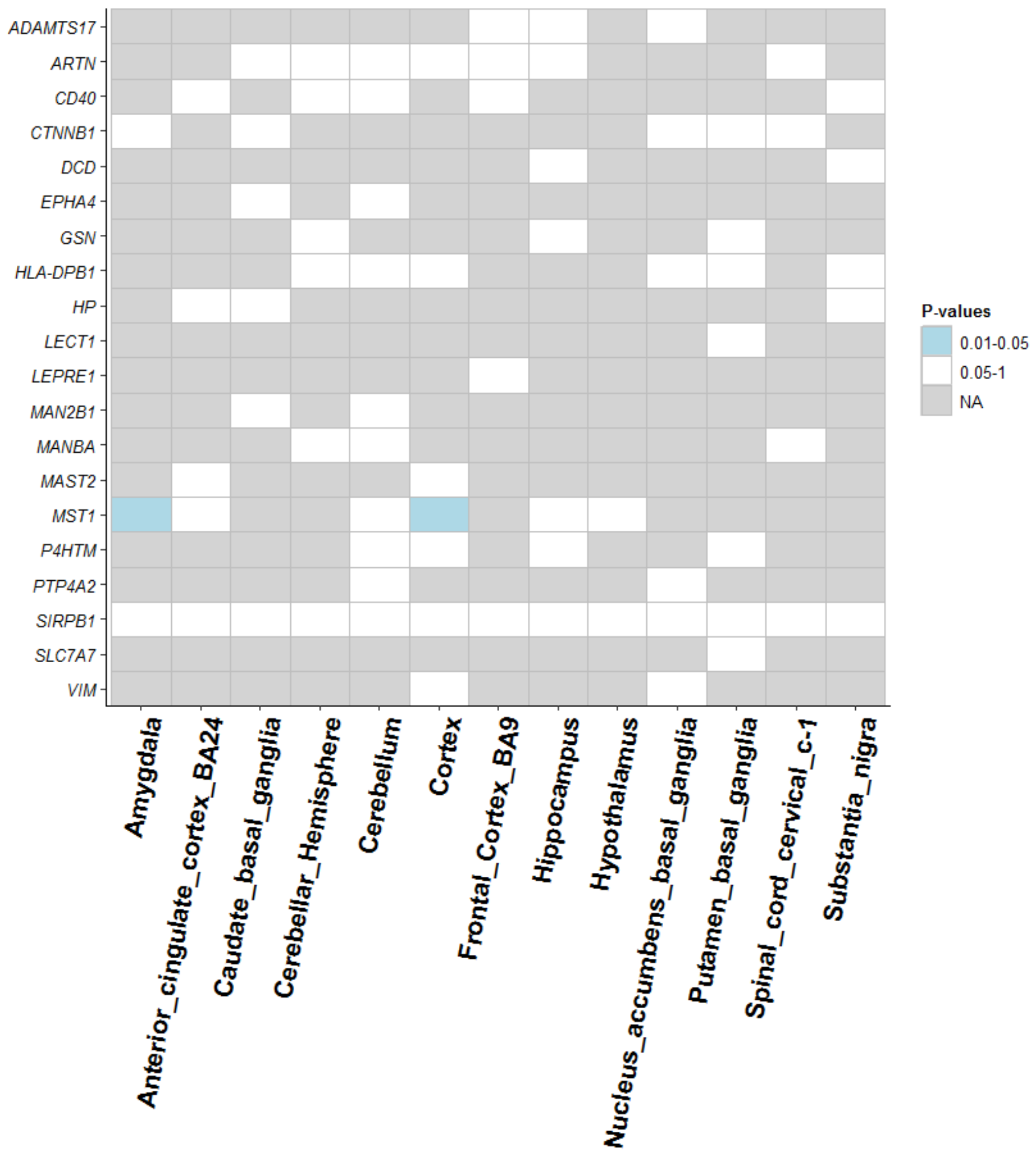

S) Subjective well-being

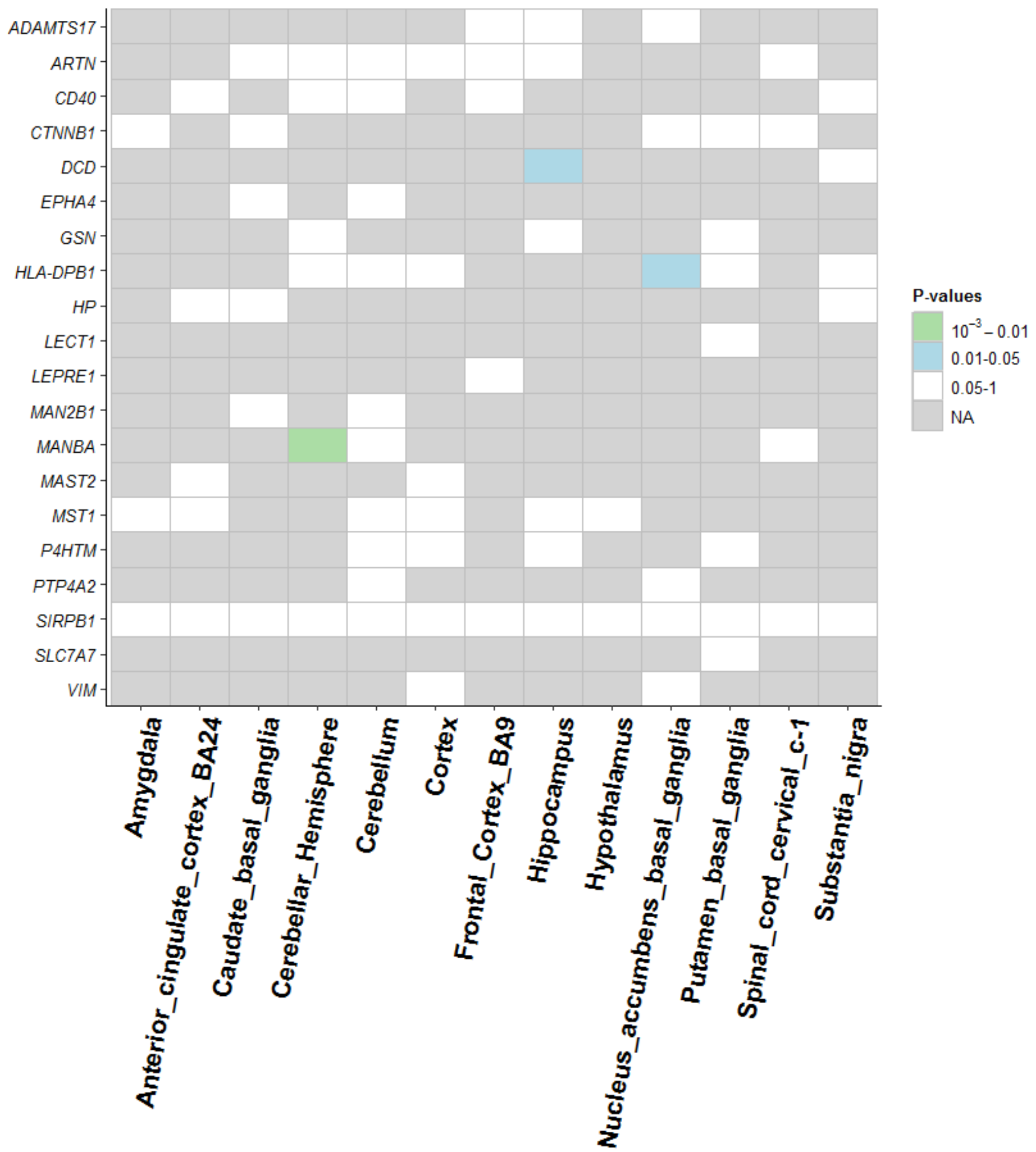

Supplement: Supplementary file 5 — Supplementary Figure 5 [file 41380_2019_540_MOESM5_ESM.pdf]
