# Supplementary material for: Druggable genome in attention deficit/hyperactivity disorder and its co-morbid conditions. New avenues for treatment
Source: Mol Psychiatry. 2019 Oct 18;26(8):4004–15. doi: 10.1038/s41380-019-0540-z (PMC7165040; doi:10.1038/s41380-019-0540-z)
Supplement: Supplementary file 16 — Supplemental Legends [file 41380_2019_540_MOESM16_ESM.docx]

Supplementary Table S1. Gene-based association between genes targeted by current FDA-approved ADHD drugs and ADHD co-morbidities.

Supplementary Table S2. Gene-set association between genes targeted by current FDA-approved ADHD drugs and ADHD as well as its co-morbidities.

Supplementary Table S3. Druggable genes associated with ADHD and their associations with the co-morbid conditions of ADHD as well as quality of life phenotypes.

Only those genes that showed association of p<0.05 in ADHD are shown. Abbreviations: ADHD; attention deficit/hyperactivity disorder, BMI; body mass index, CHD; coronary heart disease, T2DM; type 2 diabetes mellitus, SWB; subjective well-being; NPARAM, number of parameters (independent SNP signals contributing to gene’s association signal), NSNP; number of SNPs within a gene present in the respective GWA studies, ZSTAT; Z value statistic of a gene

Supplementary Table S4. Druggable genes expression level of which showed correlation with ADHD, its co-morbid conditions and phenotypes of quality of life in tissues of central nervous system.

Supplementary Table S5. Druggable GO-pathways and their associations with the co-morbid conditions of ADHD as well as quality of life phenotypes. Only those genes that showed association of p<0.05 in ADHD are shown. Abbreviations: ADHD; attention deficit/hyperactivity disorder, BMI; body mass index, CHD; coronary heart disease, T2DM; type 2 diabetes mellitus, SWB; subjective well-being, BETA: regression coefficient of a pathway; NGENE: number of genes in a pathway.

Supplementary Table S6. Druggable KEGG-pathways and their associations with the co-morbid conditions of ADHD as well as quality of life phenotypes.

Only those genes that showed association of p<0.05 in ADHD are shown. Abbreviations: ADHD; attention deficit/hyperactivity disorder, BMI; body mass index, CHD; coronary heart disease, T2DM; type 2 diabetes mellitus, SWB; subjective well-being, BETA: regression coefficient of a pathway; NGENE: number of genes in a pathway.

Supplementary Table S7. Overview of pharmacological agents targeting the druggable genes within the loci significantly associated with ADHD.

For compounds in clinical trials, the “proposed or investigated indication” is reported only for the “top 10” trials as indicated by ClinicalTrials.gov. Instances where the number of reported studies exceeds 10 are indicated with “+++”. The column titles “Approved for children?” labels the drugs that are approved for children as “Yes” and drugs that are not approved for children as “No”. Any compounds that were naturally occurring or if no information could be found, were labelled with “n/a”. This investigation did not consider off-label use of drugs in children, instead we only looked at whether the drug is FDA approved for use in children. This was done by searching for the drug in Medscape followed by a confirmation via the “Pediatric use” section of the FDA label.

Supplementary Table S8. Overview of pharmacological agents targeting the druggable genes within the loci nominally associated with ADHD (p<0.001) and significantly associated with its co-morbidities and quality of life phenotypes.

For compounds in clinical trials, the “proposed or investigated indication” is reported only for the “top 10” trials as indicated by ClinicalTrials.gov. Instances where the number of reported studies exceeds 10 are indicated with “+++”. The column titles “Approved for children?” labels the drugs that are approved for children as “Yes” and drugs that are not approved for children as “No”. Any compounds that were naturally occurring or if no information could be found, were labelled with “n/a”. This investigation did not consider off-label use of drugs in children, instead we only looked at whether the drug is FDA approved for use in children. This was done by searching for the drug in Medscape followed by a confirmation via the “Pediatric use” section of the FDA label.

Supplementary Table S9. Overview of pharmacological agents targeting the druggable genes within the loci the expression of which is significantly correlated with ADHD in tissues of central nervous system.

For compounds in clinical trials, the “proposed or investigated indication” is reported only for the “top 10” trials as indicated by ClinicalTrials.gov. Instances where the number of reported studies exceeds 10 are indicated with “+++”. The column titles “Approved for children?” labels the drugs that are approved for children as “Yes” and drugs that are not approved for children as “No”. Any compounds that were naturally occurring or if no information could be found, were labelled with “n/a”. This investigation did not consider off-label use of drugs in children, instead we only looked at whether the drug is FDA approved for use in children. This was done by searching for the drug in Medscape followed by a confirmation via the “Pediatric use” section of the FDA label.

Supplementary Table S10. Overview of pharmacological agents targeting the druggable genes within the loci the expression of which nominally correlated with ADHD (p<0.001) and significantly correlated with its co-morbidities and quality of life phenotypes in tissues of central nervous system.

For compounds in clinical trials, the “proposed or investigated indication” is reported only for the “top 10” trials as indicated by ClinicalTrials.gov. Instances where the number of reported studies exceeds 10 are indicated with “+++”. The column titles “Approved for children?” labels the drugs that are approved for children as “Yes” and drugs that are not approved for children as “No”. Any compounds that were naturally occurring or if no information could be found, were labelled with “n/a”. This investigation did not consider off-label use of drugs in children, instead we only looked at whether the drug is FDA approved for use in children. This was done by searching for the drug in Medscape followed by a confirmation via the “Pediatric use” section of the FDA label.

Supplementary Figure S1. Regional association plot of chromosome one locus.

Supplementary Figure S2. Regional association plot of chromosome four locus.

Supplementary Figure S3. Regional association plot of chromosome twelve locus.

Supplementary Figure S4. Regional association plot of chromosome three locus.

Supplementary Figure S5. Tile plot of the association between the brain expression levels of druggable genes and ADHD (p<0.001), its co-morbid conditions and quality of life phenotypes.
